# Integrating dynamic modeling of signaling pathways with subject-specific transcriptomic data to assess breast cancer risk

**DOI:** 10.1371/journal.pone.0355838

**Published:** 2026-08-13

**Authors:** Piyanut Ratphibun Yamashita, Anuwat Tangthanawatsakul, Teerasit Termsaithong, Yaowaluck Maprang Roshorm, Teeraphan Laomettachit

**Affiliations:** 1 Bioinformatics and Systems Biology Program, School of Bioresources and Technology, King Mongkut’s University of Technology Thonburi, Bangkok, Thailand; 2 School of Information Technology, King Mongkut’s University of Technology Thonburi, Bangkok, Thailand; 3 Department of Mathematics, Faculty of Science, King Mongkut’s University of Technology Thonburi, Bangkok, Thailand; 4 Mathematics and Statistics with Applications Research Group (MaSA), Faculty of Science, King Mongkut’s University of Technology Thonburi, Bangkok, Thailand; 5 Learning Institute, King Mongkut’s University of Technology Thonburi, Bangkok, Thailand; 6 Theoretical and Computational Physics Group, Center of Excellence in Theoretical and Computational Science, King Mongkut’s University of Technology Thonburi, Bangkok, Thailand; 7 Division of Biotechnology, School of Bioresources and Technology, King Mongkut’s University of Technology Thonburi, Bangkok, Thailand; 8 Pilot Plant Development and Training Institute, King Mongkut’s University of Technology Thonburi, Bangkok, Thailand; University of Vermont College of Medicine, UNITED STATES OF AMERICA

## Abstract

Breast cancer is the most common cancer in women and a leading cause of death. Traditional risk assessment models, such as the Gail model, lack molecular insight, limiting their usefulness for personalized prevention strategies. We developed a computational framework that integrates individual transcriptomic data with dynamic modeling of cell signaling to create personalized models for 30 subjects (including 15 who later developed breast cancer). Using features extracted from the dynamic simulation, we stratified individuals into four risk clusters with significantly different disease-free periods. The highest-risk group had a median disease-free period of 6.05 years, which is significantly shorter than that of the other clusters. This high-risk phenotype was characterized by hyperactive MAPK signaling (high phosphorylated ERK, phosphorylated RSK, and c-Fos). This approach demonstrates that interactions among pathway components provide additional information beyond static gene expression profiles in risk assessment and may serve as a promising tool for guiding personalized prevention strategies.

## Introduction

Breast cancer accounted for 23.8% of new cancer cases and was the leading cause of cancer-related deaths among women worldwide in 2022 [1]. Breast cancer patients in late stages (III and IV) have a lower survival rate than those diagnosed at early stages (I and II). For example, a study of young breast cancer patients aged 40 and younger found that the 5-year overall survival rate was 96.0% for stages I/II, compared to 88.3% for stage III and 64.5% for stage IV [2]. Clinical breast examination (CBE) screening has been reported to reduce the proportion of patients diagnosed with late-stage (III and IV) cancer, which fell from 47% in the unscreened group to 37% in the group that underwent four screenings and cancer awareness every two years. The screening also significantly reduced mortality by nearly 30% among women aged 50 and older [3]. Furthermore, early detection of breast cancer helps reduce the need for complex and intensive treatments in advanced stages, lowering healthcare costs [4]. Therefore, implementing effective risk stratification methods is crucial for developing personalized screening and prevention strategies, which can facilitate early detection and ultimately improve survival rates among breast cancer patients.

To support these efforts, several risk assessment models have been developed to estimate an individual’s likelihood of developing breast cancer and guide clinical decision-making. Among these, the National Cancer Institute’s Breast Cancer Risk Assessment Tool (BCRAT or Gail Model) [5], the Breast Cancer Surveillance Consortium (BCSC) model [6], and the Tyrer-Cuzick model [7] are commonly used. These models depend on compiling a person’s clinical and reproductive history, such as age at menarche, childbirth history, biopsy results, and family history of breast cancer, to determine a risk score. The Gail Model was one of the earliest breast cancer risk assessment tools. It uses basic personal factors and first-degree family history to estimate invasive risk, making the model the most highly accessible. The BCSC model improves upon the Gail model by integrating breast density as a crucial risk factor, making it highly valuable for women undergoing routine screening. The Tyrer-Cuzick model incorporates *BRCA1/2* genetic status, detailed family history (up to third-degree relatives), and breast density to estimate risk for both invasive and non-invasive breast cancer.

Although traditional risk assessment models are useful, they rely on population-level epidemiological data and often lack the sensitivity and specificity needed for predicting individual outcomes. They also have significant limitations in offering a personalized and mechanistic understanding of disease initiation. Alternatively, transcriptomics is being investigated for risk assessment by examining transcriptomic profiles of non-malignant breast tissue to identify molecular signatures associated with susceptibility.

Research indicates that gene expression in patient-derived, benign-appearing breast tissue samples adjacent to breast tumors displays distinct and prognostically relevant transcriptomic subtypes [8,9]. Román-Pérez et al. (2012) first established this by identifying two distinct transcriptomic subtypes, Active and Inactive, in cancer-adjacent benign-appearing tissue. The Active subtype was characterized by elevated expression of genes involved in cellular movement and fibroblast activation, and shared distinct features with the claudin-low breast cancer subtype, notably reduced expression of cell adhesion and cell-cell contact genes. The Active subtype conferred a hazard ratio of approximately 2.5–2.6 for worse overall survival, specifically among ER-positive and hormone-treated patients [8]. This was subsequently validated by Troester et al. (2016) using TCGA data, in which the same two transcriptomic clusters were reproduced in a large multi-institutional cohort, and the Active subtype remained significantly associated with worse 10-year survival among ER-positive patients in multivariate analysis [9].

Critically, subsequent work demonstrated that this risk-associated Active transcriptome phenotype is not restricted to cancer-adjacent tissue but is detectable in histologically normal breast tissue from healthy women. Kang et al. (2020) found that over 50% of breast tissue samples from healthy donors with no history of breast disease displayed the Active transcriptome phenotype [10]. Within this cohort, donors expressing the Active transcriptome phenotype had significantly higher Gail risk scores than those with the Inactive phenotype, providing a direct link between the transcriptomic phenotype and an established clinical risk metric. Notably, most subjects from whom the samples were taken had Gail scores below the clinical eligibility threshold and were not qualified for endocrine prevention therapy, suggesting that transcriptomic profiling may identify at-risk individuals who would otherwise go undetected by conventional risk assessment [10].

In another study, expression profiling of normal breast tissues from healthy women has identified a tissue subtype that shares the same molecular features as the claudin-low breast cancer phenotype, including downregulation of epithelial markers and enrichment of stem cell-like and mesenchymal characteristics [11]. The presence of this stem-like signature in histologically normal tissue suggests a baseline enrichment of cells with high plasticity and dedifferentiation potential, which may be associated with a high susceptibility to oncogenic transformation [11].

Crucially, these findings demonstrate that histologically normal breast tissue may contain early molecular indicators of susceptibility before any clinical or morphological changes, therefore justifying the use of normal tissue transcriptomics to assess and predict future breast cancer risk.

While transcriptomic profiling is a promising method that provides valuable molecular insights, these profiles alone offer only limited information from static snapshots of gene expression. They fail to account for the complex and dynamic interactions and post-translational regulation within critical signaling pathways, which may underlie disease progression and risk. To gain a deeper understanding of the interactions between genes and proteins in signaling pathways, transcriptomic data have been integrated with mathematical models. This process uncovers biological mechanisms contributing to disease in individuals by using individual patient data to personalize the models, such as modifying variable activity status, kinetic rates, and initial conditions [12–14]. Integrating personal biological data into the models enables the development of ‘digital twins,’ virtual representations of individuals designed to provide tailored healthcare and personalized medicine [15–20]. For example, Imoto et al. (2022) [13] utilized gene expression data from individual patients to simulate signaling networks and predict patient outcomes. The study focused on the ErbB receptor pathway in breast cancer, classifying patients into prognostic groups based on the dynamics of the signaling pathways. It identified patterns in these signaling dynamics that were associated with resistance to ErbB-targeted therapies.

In this study, we focus on assessing breast cancer risk through a personalized method that combines gene expression data with dynamic modeling of signaling pathways. By incorporating the transcriptomes of 30 healthy women into a signaling pathway model and extracting features from dynamic simulations, we can stratify subjects into groups with different levels of susceptibility to breast cancer development. This new approach provides effective risk predictions and enhances understanding of the underlying processes that may lead to breast cancer, highlighting further potential for preventive strategies.

## Results

### Overview of the framework

This study presents a personalized dynamic modeling approach to assess breast cancer risk by integrating transcriptomic data with mathematical simulations of cellular signaling pathways. We began by developing a mathematical model based on ordinary differential equations (ODEs) to simulate the interactions between proteins and genes in the proliferation and apoptosis signaling pathways. The model then incorporated transcriptomic data from breast tissue samples donated by 30 women, who were considered healthy at the time of donation; however, 15 of them were later diagnosed with breast cancer. Individual gene expression profiles were used to personalize model parameters, enabling us to simulate dynamic profiles for each person. We then extracted features from the dynamic simulations, which were used to stratify subjects into distinct risk groups. This process improves our understanding of breast cancer susceptibility and has the potential to develop into a tool for assessing the risk of breast cancer development.

### Constructing a mathematical model to simulate the interactions between proteins in the proliferation and apoptosis pathways

**Fig 1:**
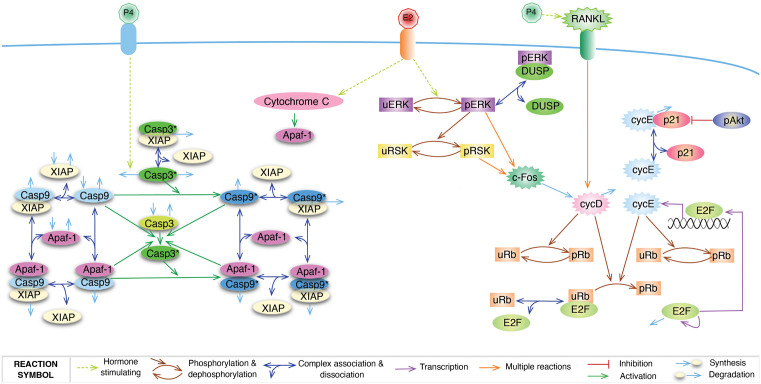
Interaction diagram of components in the proliferation and apoptosis pathways. Arrows represent reactions or signaling interactions described by kinetic rates in the ODE model (S2 and S3 Tables in S1 Text).

We constructed an ordinary differential equation (ODE) model that simulates the complex interactions between components regulating proliferation and apoptosis pathways (Fig 1). Two previously published models were chosen as the starting point for our model. The first model by Imoto & Okada [21] focused on proliferation regulation, while the second model by Legewie et al. [22] addressed the apoptosis pathway. We adopted the components from these models, which include ERK, DUSP, RSK, c-Fos, cyclin D, RB, E2F, cyclin E, p21 in the proliferation pathway [21] and cytochrome c, caspase-3, and caspase-9 in the apoptosis pathway [22]. Table 1 lists the components in the model, and S2 and S3 Tables, in S1 Text, provide the equations of the model and the description of each variable.

**Table 1:** Descriptions of genes and proteins in the model.

Gene name	Protein name	Protein description	Related components in the model
*MAPK1*	ERK2	Extracellular signal-regulated kinase 2	[pERK], [pERK:DUSP]
*DUSP5*	DUSP5	Dual specificity phosphatase 5	[pERK:DUSP]
*RPS6KA2*	RSK3	p90 ribosomal S6 kinase 3	[pRSK]
*FOS*	c-Fos	Protein c-Fos	[c-Fos]
*TNFSF11*	RANKL	Receptor activator of nuclear factor-κB ligand	[RANKL]
*CCND1*	Cyclin D	Cyclins D (D1, D2, and D3)	[CycD]
*RB1*	RB	Retinoblastoma protein	[uRB]
*E2F1*	E2F1	E2 promoter binding factor 1	[tE2F], [E2F]
*CCNE1*	Cyclin E	Cyclins E	[tCycE], [CycE:p21]
*CDKN1A*	p21	Potent cyclin-dependent kinase inhibitor	[CycE:p21]
*AKT1*	AKT	Ak strain transforming	[pAKT]
*SKP2*	SKP2	S phase kinase-associated protein 2	[SKP2]
*CYCS*	Cytochrome c	Cytochrome c	[CytoC], [CytoC:Casp9], [CytoC:Casp9*], [CytoC:Casp9:X], [CytoC:Casp9*:X]
*CASP9*	Caspase-9	Caspase-9	[Casp9], [Casp9*], [Casp9:X], [Casp9*:X], [CytoC:Casp9], [CytoC:Casp9*], [CytoC:Casp9:X], [CytoC:Casp9*:X]
*CASP3*	Caspase-3	Caspase-3	[Casp3], [Casp3*], [Casp3*:X],

To capture the dynamical behaviors of the pathways in response to external factors, the influences of the two ovarian hormones, estrogen (E2) and progesterone (P4), were incorporated into the model. Both E2 and P4 are key regulators of proliferation and apoptosis pathways (Fig 1). E2 involves upstream signal transduction through Extracellular Signal-Regulated Kinase (ERK) [23], which is encoded by the *MAPK1* gene. E2 also functions upstream of cytochrome c [24], stimulating apoptosis. P4 signaling induces the secretion of RANKL from PR+ cells, which acts in a paracrine manner on neighboring RANK+ progenitors, activating the NF-κB pathway to upregulate cyclin D1 levels and promote proliferation [25,26]. In contrast, P4 inhibits apoptosis by deactivating caspase-3 [27]. The periodic function has been used to explain the periodic oscillation in both E2 and P4 (see S1 Text).

### Exploring breast tissue transcriptome

**Fig 2:**
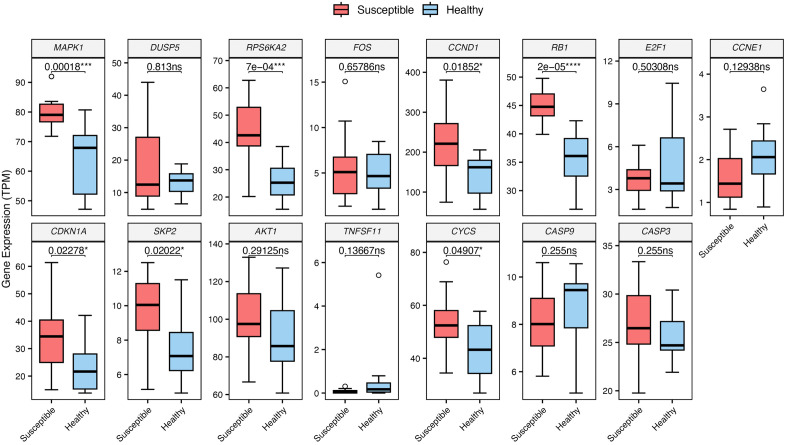
Comparison of gene expression levels in the TPM normalization unit between the healthy (subjects whose latest record was still disease-free) (blue) and the susceptible (subjects who had later been diagnosed with breast cancer) (red) groups. Data were retrieved from Gene Expression Omnibus (GSE166044). The p-value is calculated using the non-parametric Wilcoxon test and adjusted using the Benjamini-Hochberg (FDR) method. The asterisks indicate p.adj ≤ 0.0001 (****), p.adj ≤ 0.001 (***), p.adj ≤ 0.01 (**), and p.adj ≤ 0.05 (*). ns: not significant.

Whole transcriptome data from 30 healthy women at the time of donation were retrieved from the GEO database (GSE166044). We categorized the samples into two groups: 15 subjects whose latest record was still disease-free (healthy group) and 15 subjects who had later been diagnosed with breast cancer (susceptible group). Fig 2 compares the expression profiles of the genes corresponding to the components in our model between healthy and susceptible groups. *MAPK1* (encoding ERK2), *RPS6KA2* (encoding RSK3), *CCND1* (encoding cyclin D), *RB1* (encoding RB), *CDKN1A* (encoding p21), and *SKP2* (encoding SKP2) levels were significantly higher in the susceptible group. Additionally, the susceptible group showed higher *CYCS* gene expression (encoding cytochrome c), although the statistical evidence for the difference was less marked (adjusted p-value of 0.0491). S5 Table presents the complete list of p-values and effect sizes.

### Integrating gene expression into the dynamic model

We refer to the model described in S2 and S3 Tables in S1 Text as the generic model of breast tissue proliferation and apoptosis pathways. We personalized the generic model into individualized models by adjusting its parameters based on each individual’s gene expression profile. Two groups of parameters were adjusted: 1) parameters representing the total concentration of the proteins and 2) parameters representing the synthesis rate of the proteins. For variables whose activities are controlled at the post-translational level, such as protein modification and stoichiometric inhibition (e.g., ERK, RSK, and RB), individual gene expression levels were used to adjust the total protein amount in the individualized models. For variables whose activity is mainly controlled by synthesis and degradation (e.g., c-Fos and CycD), individual gene expression levels were used to adjust the protein’s synthesis rate.

For parameters personalized via synthesis rates, the rate of protein production is scaled proportionally to the individual’s gene expression level relative to the healthy population mean. In other words, a subject’s protein synthesis rate in the model will be higher or lower depending on whether their individual gene expression is higher or lower than the healthy control average.

For parameters personalized via total concentrations, the overall protein level is scaled proportionally to the individual’s gene expression compared to the healthy population average. Essentially, a subject’s initial pool (total concentration) of the naive (e.g., unphosphorylated) protein form in the model will be higher or lower depending on whether their gene expression exceeds or falls below the healthy control mean. For instance, a person with elevated *MAPK1* expression begins with a larger baseline pool of unphosphorylated ERK. This expansion enhances the system’s maximum signaling capacity. As a result, when stimulated by hormonal oscillations, a person with higher *MAPK1* expression will produce more active, phosphorylated ERK (pERK), thereby speeding up downstream signaling processes (e.g., the conversion of inactive RSK to active pRSK).

These parameter adjustments reflect the underlying biology. While transcript levels inform baseline protein abundance, the actual signaling activity is further shaped by post-translational modifications. Overall, the core concept of our methodology is that transcriptomic data provide a static snapshot of transcript abundance, and the ODE model translates these static inputs into dynamic, post-translationally regulated activation profiles (such as pERK and pRSK) via kinetic interactions and hormone-driven rate equations.

To implement the concept discussed above, we first calculated a scaling factor ω, which is the ratio between the parameter values from the original models [21,22] (*y*_n_ in Table 2) and the average gene expression level of the healthy group (*y*_a_ in Table 2 and also the blue bars in Fig 2). The scaling factor (ω = *y*_n_/*y*_a_) was then used to scale the parameter values for all individuals in the dataset (both healthy and susceptible). For example, the personalized model of an individual whose *MAPK1* expression level (in the TPM normalization unit) is 70 will have the total concentration of the ERK protein, [tERK], in the model equal to ω_ERK_ × 70 = 1.133 × 70 = 79.31, and the personalized model starts with unphosphorylated ERK at 79.31. Table 2 lists the parameters that are personalized in the individual models along with their scaling factor (ω) values.

**Table 2:** The parameters that are personalized in the model based on the gene expression profiles of individuals.

Gene name	Personalized parameters	$y_{n}$	$y_{a}$	$\omega =\frac{y_{n}}{y_{a}}$
*AKT1*	[pAKT]	1.0	91.965	0.011
*CASP3*	*k*_syn-C3_	12.0	25.654	0.468
*CASP9*	*k*_syn-C9_	1.2	8.721	0.138
*CCND1*	*k*_syn-CycD-cFos_	0.005	138.6189	0.00003
	*k*_syn-CycD-RANKL_	16.8	138.6189	0.121
*CCNE1*	*k*_syn-CycE-E2F_	0.01	2.276	0.004
*CDKN1A*	[tp21]	1.5	22.779	0.066
*CYCS*	*k*_act-CytoC-E2_	0.011	42.564	0.0003
*DUSP5*	[tDUSP]	1.0	14.519	0.069
*E2F1*	*k*_syn-E2F_	0.03	4.968	0.006
	*k*_syn-E2F-E2F_	0.04	4.968	0.008
*FOS*	*k*_syn-cFos_	0.006	6.958	0.001
*MAPK1*	[tERK]	72.36	63.863	1.133
*RB1*	[tRB]	5.0	35.610	0.14
*RPS6KA2*	[tRSK]	0.8	26.168	0.031
*SKP2*	[SKP2]	1.0	8.192	0.122
*TNFSF11*	*k*_syn-RANKL_	0.39	2.854	0.137

### Simulating personalized models revealing diverse protein dynamics profiles across individuals

**Fig 3:**
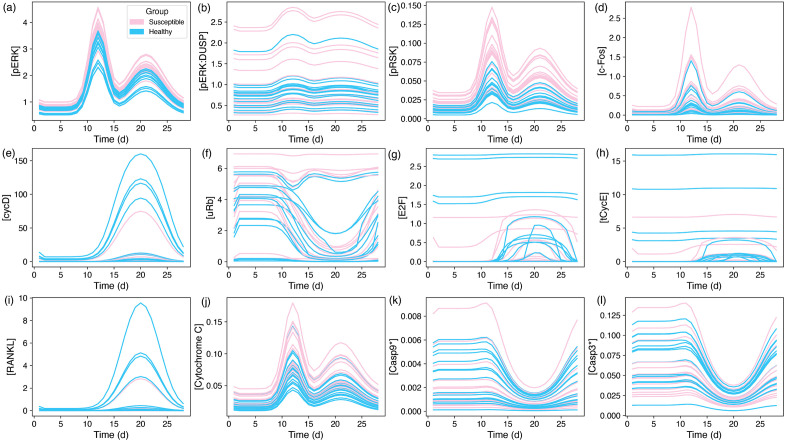
The dynamic simulations of 30 individualized models. Fifteen healthy controls and fifteen susceptible subjects are represented in blue and pink, respectively. (a) pERK, (b) pERK:DUSP complex, (c) pRSK, (d) c-Fos, (e) cyclin D, (f) uRB, (g) E2F, (h) cyclin E, (i) RANKL, (j) cytochrome c, (k) Casp9*, (l) Casp3*.

Fig 3 displays the dynamic simulations of individuals from the 30 personalized models. Since pERK, pRSK, c-Fos, and cytochrome c are downstream targets of E2, they show two peaks following fluctuations in E2 on days 11 and 20 (Fig 3a, 3c, 3d, and 3j). P4 reaches its highest point on day 19, leading to an increase in RANKL and cyclin D (Fig 3i and 3e), and a decrease in active caspase-3 (Casp3*) and the unphosphorylated form of RB (uRB) (Fig 3l and 3f).

Simulations of individual subjects reveal interesting patterns related to their health status. First, the healthy and susceptible groups are distinctly separated based on the profiles of pERK, pRSK, and cytochrome c (Fig 3a, c, and j). This separation aligns with the expression levels of their respective genes—*MAPK1*, *RPS6KA2*, and *CYCS*—which are identified as differentially expressed genes in Fig 2.

In contrast, some components with differentially expressed genes in Fig 2, such as *RB1*, show their post-translational forms (e.g., unphosphorylated RB, uRB) displaying mixed trajectories between healthy and susceptible groups (Fig 3f). While *RB1* gene expression indicates the potential protein level, the actual function of the RB protein is mainly regulated post-translationally by cyclin D and cyclin E. Although *RB1* expression is statistically higher in the susceptible group (Fig 2), dynamic modeling reveals variable uRB trajectories within this group, suggesting biologically relevant subgroups. This suggests that the profiles of components involved in complex interactions may provide additional information beyond that accessible from gene expression data alone. The diverse responses observed through dynamic modeling of individuals may offer significant potential for stratifying subjects based on their simulation profiles, as demonstrated shortly.

### Extracting features from the dynamic model simulation

**Fig 4:**
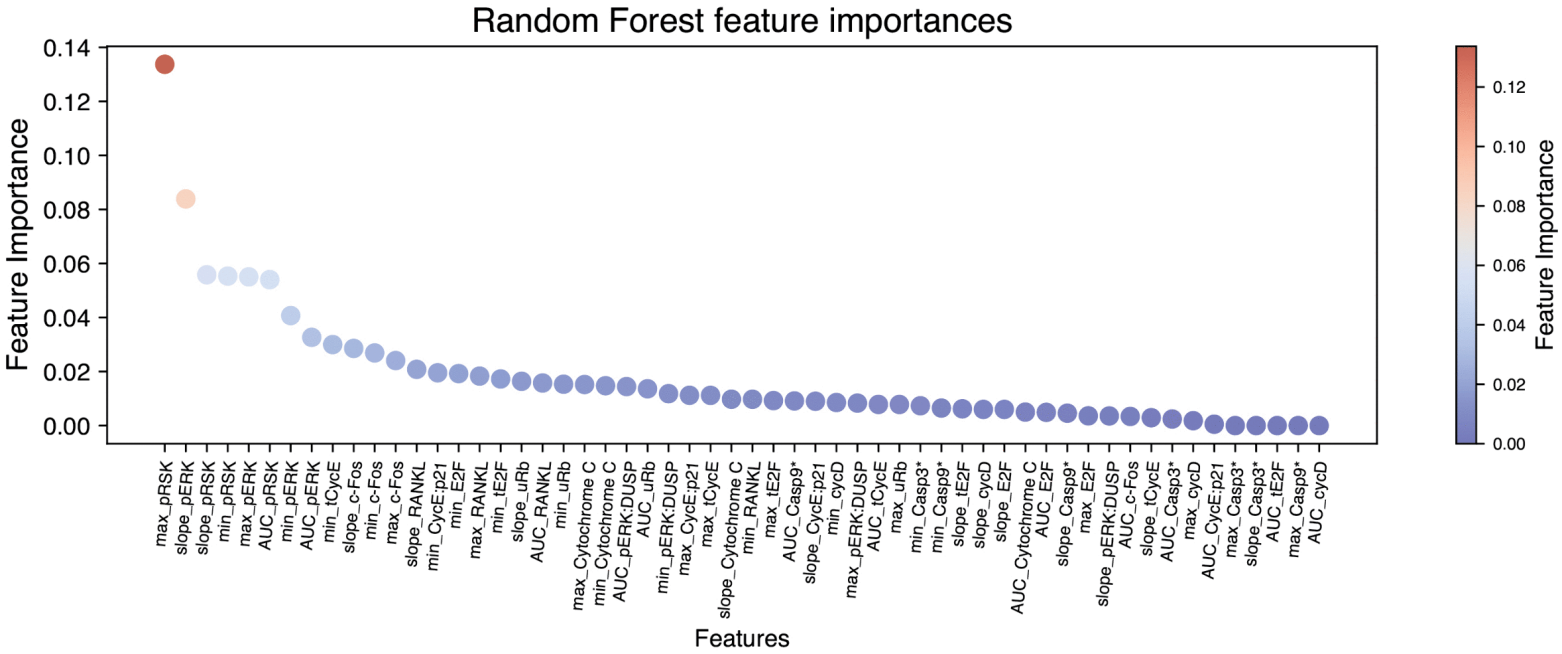
Features extracted from dynamic simulations are ranked based on importance scores from a Random Forest model.

To utilize individual simulation profiles for stratifying subjects into groups with meaningful susceptibility interpretation, we calculated four types of features extracted from the dynamics of each variable: (i) cumulative protein activity or area under the curve (AUC), (ii) maximum activity (max), (iii) minimum activity (min), and (iv) rate of activity change (absolute slope between the max and min values). We computed the four features from each of the following variables: [pERK], [pERK:DUSP], [pRSK], [c-Fos], [CycD], [uRb], [tE2F], [E2F], [tCycE], [CycE:p21], [RANKL], [CytoC], [Casp3*], and [Casp9*], resulting in a total of 56 features. To select only relevant features for further analysis, we ranked them based on their importance scores, calculated using the Gini index criterion within a Random Forest model that classified subjects into healthy and susceptible groups (Fig 4).

### Stratifying individuals into clusters with varying risk

We utilized features extracted from dynamic simulation to stratify subjects into clusters while varying several clustering parameters, including the number of top features (from top 10–56), the number of clusters (*k* from 2 to 6), the distance method (Binary, Canberra, Euclidean, Manhattan, Maximum, and Minkowski), and the hierarchical clustering method (Average, Centroid, Complete, McQuitty, Median, Single, Ward.D, and Ward.D2). Then, the disease-free survival curve for each cluster was estimated using the Nelson-Aalen derived survival function. Next, the Fleming-Harrington test was conducted to determine the p-value for the overall difference in disease-free distributions among the clusters. The parameter sets that produced the following results were filtered out: p-values > 0.05, any cluster with < 3 members, and any cluster whose median disease-free year has not been reached (presumably the healthy subject clusters) having a follow-up span of less than 10 years. The last requirement was to ensure that the group whose median disease-free year has not yet been reached is genuinely due to a long disease-free period, not to insufficient observation time. The parameter sets that passed the criteria were further evaluated using the Silhouette coefficient, which measures the similarity of a subject to their own cluster compared to other clusters. Table 3 displays five parameter sets with the highest Silhouette coefficients.

**Table 3:** Results of clustering analysis from five different parameter sets with the highest values of the Silhouette coefficient.

Parameter set	Number of top features used	Number of clusters (*k*)	Distance method	Hierarchical clustering method	Median disease-free year for each cluster	Number of subjects in each cluster	p-value	Silhouette coefficient	Error
1	20	4	maximum	ward.D	NA, 10.59, 9.08, 7.48	10, 7, 6, 7	0.023	0.289	3.48
2	26	4	canberra	complete	NA, 10.59, 9.08, 6.05	11, 5, 5, 9	0.006	0.227	2.38
3	26	5	canberra	complete	NA, 10.59, 9.08, 6.05, 7.48	11, 5, 5, 4, 5	0.013	0.225	2.45
4	31	4	canberra	complete	NA, 10.59, 9.08, 6.05	11, 5, 5, 9	0.006	0.205	2.38
5	31	5	canberra	complete	NA, 10.59, 9.08, 6.05, 7.48	11, 5, 5, 4, 5	0.013	0.202	2.45

NA indicates the median disease-free year has not been reached.

Next, we further evaluated the performance of the five parameter sets by calculating the error between the actual disease-free year for each susceptible subject and the median disease-free year for the respective cluster estimated from the Nelson-Aalen derived survival function. Table 4 demonstrates the calculation of the error for Parameter Set 2. The error determines how accurately the estimated median disease-free periods predict breast cancer risk. The deviation of the actual disease-free periods from the estimated median values was calculated as $\sqrt{\frac{\sum {\text{Diff}}^{\text{2}}}{N}}$, where Diff is the year difference between the actual and estimated disease-free periods. This calculation includes only the susceptible subjects assigned to Clusters 2–4 (*N* = 13), and the result showed an error of 2.38 years (Table 4).

**Table 4:** Comparison between the actual and predicted disease-free periods and the resulting prediction error for the 15 susceptible individuals.

Subject ID	Assigned cluster	Lapse years from the first donation to cancer diagnosis	Median disease-free years of the cluster	Year difference (Diff)
K106357	4	6.05	6.05	0
K107977	4	4.93	6.05	−1.12
K104653	3	9.08	9.08	0
K104589	4	7.92	6.05	1.87
K105140	4	7.48	6.05	1.43
K107273	1	3.54	NA	NA
K108358	2	4.02	10.59	−6.57
K104430	4	7.76	6.05	1.71
K107138	4	8.59	6.05	2.54
K107237	4	3.54	6.05	−2.51
K107912	4	4.48	6.05	−1.57
K104728	2	10.76	10.59	0.17
K105316	4	3.65	6.05	−2.4
K106859	1	4.76	NA	NA
K107616	2	10.59	10.59	0
				$\sqrt{\frac{\sum {\text{Diff}}^{\text{2}}}{N}}$ = 2.38 years

NA was excluded from the error calculation.

The same calculation was used to determine the error for each parameter set (Table 3). This method identified Parameter Sets 2 and 4 as having the lowest error of 2.38 years (Table 3). Parameter Sets 2 and 4 used the same distance and hierarchical clustering method and yielded the same four clusters with the same subjects. Therefore, we chose Parameter Set 2 as it used fewer features (26) than Parameter Set 4 (31).

**Fig 5:**
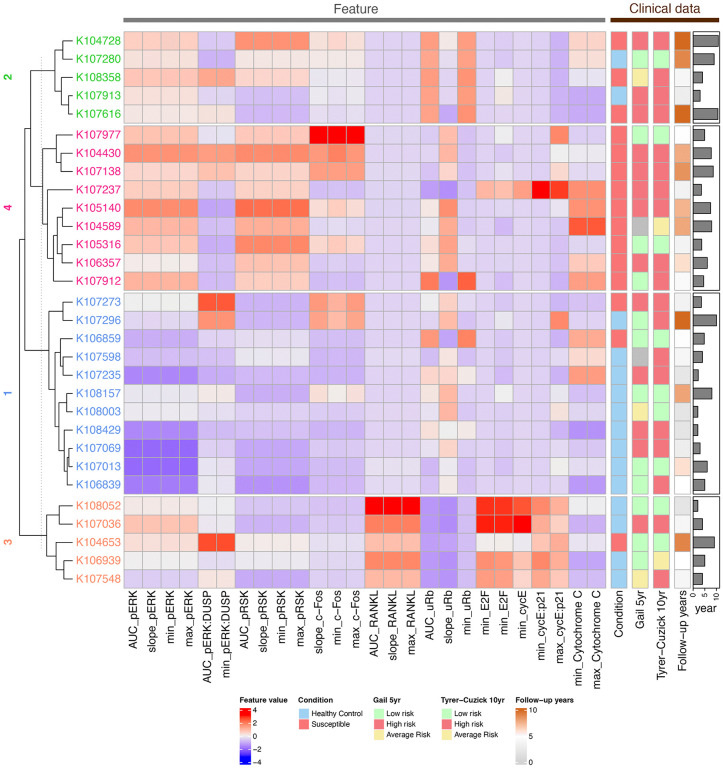
Heatmap of 26 features extracted from dynamic simulations of 30 subjects, clustered based on Parameter Set 2 in Table 3.

**Fig 6:**
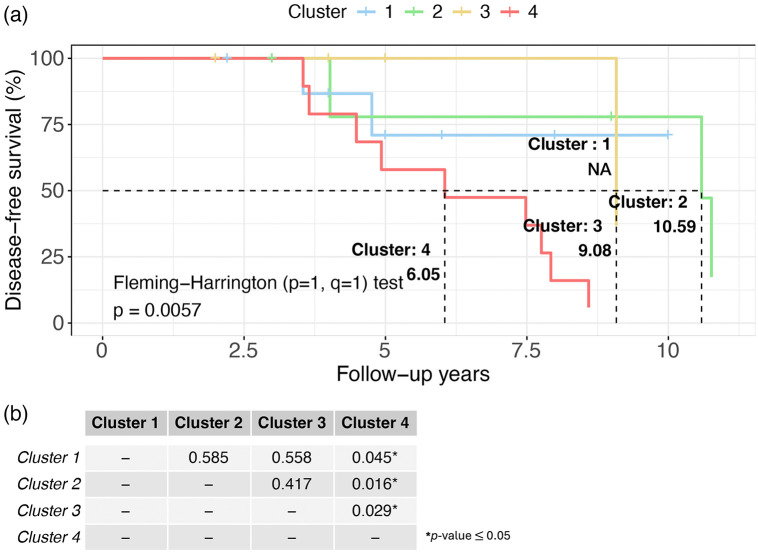
Disease-free survival curves. (a) Disease-free survival curves for the four clusters. Survival probabilities were estimated using the non-parametric survival function derived from the Nelson-Aalen estimator. The difference in disease-free survival across all four clusters was assessed using the Fleming-Harrington (ρ = 1, ɣ = 1) weighted log-rank test. Cluster 1 did not reach the median within the follow-up period. (b) Pairwise comparison of disease-free survival between the four clusters. Asterisks (*) indicate a statistically significant difference in disease-free survival (p-value ≤ 0.05).

We next present the clustering analysis results in detail for Parameter Set 2 (26 top features with four clusters, using the Canberra distance method and the Complete hierarchical clustering method). Hierarchical clustering using Parameter Set 2 stratified the cohort into four distinct clusters (Fig 5). Cluster 1 contains 11 members, 9 of whom are from the healthy group. The median disease-free period for Cluster 1 had not been reached because most subjects remained disease-free as of the latest reported status. The median disease-free periods for Clusters 2, 3, and 4 are 10.59, 9.08, and 6.05 years, respectively (Fig 6a). Notably, Cluster 4, with the lowest median disease-free period of 6.05 years, is characterized by elevated activation of pERK, pRSK, c-Fos, and consists entirely of subjects from the susceptible group (Fig 5). A statistical comparison reveals that the disease-free period of cluster 4 was significantly different from that of the other clusters (p < 0.05; Fleming-Harrington test) (Fig 6b), suggesting that the simulation profiles of subjects in this cluster may be a reliable indicator for early breast cancer risk assessment. More interestingly, despite the confirmed high risk of Cluster 4, the clinical risk assessment shows several subjects in this cluster have low Gail 5-year and Tyrer-Cuzick 10-year risk scores (Fig 5), indicating that traditional risk models may fail to identify this specific subset of high-risk subjects.

### Interactions between components providing further biological information for breast cancer risk assessment

Next, we examine our modeling simulation results to gain insights into the biological features that may drive disease development and progression within each cluster. We emphasize that our modeling approach captures the dynamic interactions among various components. This method offers additional information about proteins that extends beyond what gene expression data alone can provide, potentially allowing for more effective stratification of subjects into distinct clusters.

### Interactions between pERK, pRSK, and c-Fos as important indicators for breast cancer risk stratification among the four clusters

The MAPK pathway is a key component in cellular processes that promote cancer [28–30]. ERK functions downstream of the MAPK pathway and is activated by phosphorylation. In breast cancer, ERK activity is often elevated, resulting in uncontrolled cell growth [31]. RSK is a direct target of ERK [32,33] and plays a crucial role in controlling protein synthesis and cell growth. c-Fos, a downstream target of the MAPK pathway [34], is a transcription factor that activates genes involved in cell proliferation, survival, and differentiation [35]. Overexpression of *FOS* is frequently observed in various cancers and is linked to a more aggressive phenotype [36–39].

**Fig 7:**
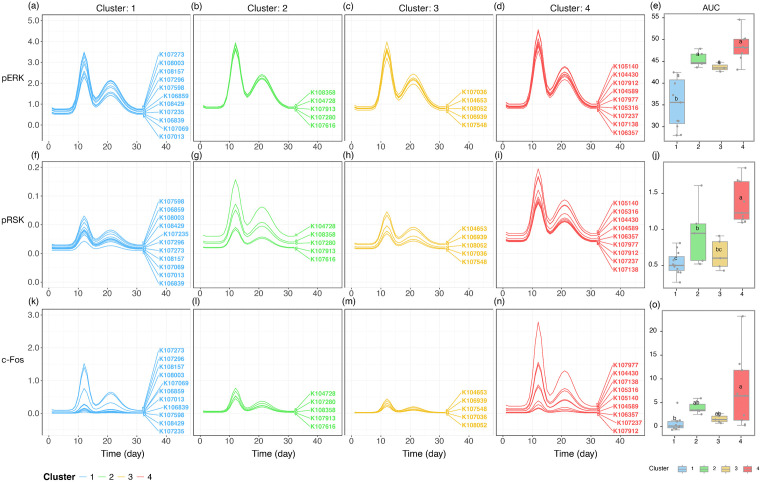
Dynamic simulations of 30 individual subjects stratified into four clusters. (a-e) pERK (f-j) pRSK (k-o) c-Fos. Bars labeled with different letters are significantly different from one another, as assessed by Tukey’s HSD test after a one-way ANOVA (p < 0.05).

Our feature selection analysis from the previous section showed that the phosphorylated forms of ERK (pERK) and RSK (pRSK), and c-Fos were among the most important features (Fig. 4). Model simulations of pERK activity (Fig 7a-e) aligned with *MAPK1* gene expression (Low in Cluster 1; High in Clusters 2–4) (S1 Fig., panel a, in S1 Text), as the expression values were incorporated into our individualized models as the total amount of ERK protein.

However, downstream signaling revealed more complex dynamics. Specifically, the model calculated pRSK activity by integrating total RSK levels (from *RPS6KA2* expression) with pERK activation (from our model simulation). This produced a distinct activity gradient: Low (Cluster 1), Medium (Clusters 2 and 3), and High (Cluster 4) (Fig 7f-j). Most notably, our dynamic simulation uncovered hidden stratification in c-Fos levels. While *FOS* gene expression from transcriptomic data was similar across all clusters, with no statistically significant differences detected (S1 Fig., panel d, in S1 Text), the model predicted divergent c-Fos protein levels driven by the co-regulation of pERK and pRSK (Fig 7k-o). This dynamic simulation enabled stratification of clusters into risk categories corresponding to clinical outcomes: Low risk in Cluster 1 (median disease-free period not reached), Medium in Clusters 2 and 3 (10.59 and 9.08 years, respectively), and High in Cluster 4 (6.05 years).

### Interactions between RANKL, Cyclin D, RB, and E2F1 as important indicators for breast cancer risk stratification of Cluster 3

**Fig 8:**
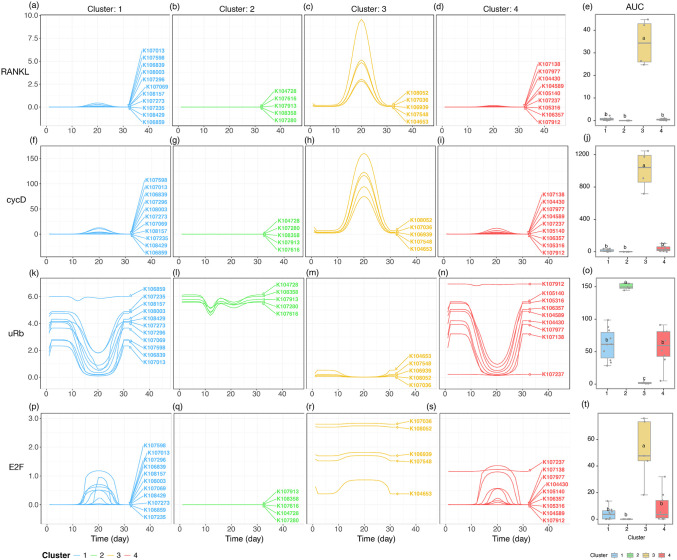
Dynamic simulations of 30 individual subjects stratified into four clusters. (a-e) RANKL (f-j) CycD (k-o) uRB (p-t) E2F1. Bars labeled with different letters are significantly different from one another, as assessed by Tukey’s HSD test after a one-way ANOVA (p < 0.05).

Another critical interaction involves the RANKL → Cyclin D –| RB –| E2F1 signaling axis. While *RB1* gene expression varied inconsistently across clusters (S1 Fig., panel f, in S1 Text), our dynamic modeling revealed a functional convergence for Clusters 1, 2, and 4. In these clusters, low levels of upstream RANKL and Cyclin D signaling (Fig 8a-j) resulted in substantial levels of unphosphorylated, active RB (uRB) (Fig 8k-o). This active uRB functions as an inhibitor, suppressing E2F1 levels (Fig 8p-t) and inhibiting cell cycle progression [40].

In contrast, Cluster 3 exhibited a distinct mechanistic pattern. With moderate *RB1* expression (S1 Fig., panel f, in S1 Text) and high expression levels of upstream RANKL (S1 Fig., panel l, in S1 Text) from the transcriptome data, our simulation predicted that RB was effectively inactivated (resulting in low uRB) (Fig 8m). This loss of RB inhibition led to elevated E2F1 activity (Fig 8r), the master regulator of cell cycle progression [41,42]. This specific pathway distinguishes Cluster 3 from the others and may provide a mechanistic explanation for why Cluster 3 subjects experience a shorter disease-free period than those in Clusters 1 and 2.

Table 5 summarizes the molecular characteristics of each cluster that may underlie the observed development of breast cancer. Cluster 1, representing the lowest-risk group that did not reach the median disease-free survival during the follow-up period, is characterized by low levels of all three key oncogenic proteins in the MAPK pathway (pERK, pRSK, and c-Fos). Conversely, Cluster 4, classified as the high-risk group, exhibits the shortest median disease-free survival of 6.05 years and is characterized by a hyperactive MAPK signaling profile (high pERK, pRSK, and c-Fos). Clusters 2 and 3 represent intermediate-risk groups (median disease-free survival of 10.59 and 9.08 years, respectively) but show subtle differences in their molecular profiles. Both clusters show high pERK levels but intermediate RSK and c-Fos levels, suggesting less intense activation of the MAPK signaling cascade than in the high-risk Cluster 4. However, the key distinction is that Cluster 3 displays a strong cell cycle dysregulation profile (High RANKL/CycD, Low uRB, High E2F), making it highly proliferative, which likely accounts for its shorter disease-free survival compared to Cluster 2.

**Table 5:** Summary of the simulation profile and the corresponding predicted risk level for each cluster.

Cluster	Median year	pERK	pRSK	c-Fos	RANKL/CycD	uRB	E2F1	Cancer signal profiles	Risk level
1	Not reached	Low	Low	Low	Low	Med	Low	–	Low
2	10.59	High	Med	Med	Low	High	Low	High pERK	Med
3	9.08	High	Low/Med	Med	High	Low	High	High pERK, Low uRB, High E2F1	Med
4	6.05	High	High	High	Low	Med	Low	High pERK, High RSK, High c-Fos (Hyperactive MAPK)	High

### Dynamic modeling revealing enhanced caspase activity in Cluster 3 beyond transcript levels

Additionally, although caspase-9 and caspase-3 were not among the top selected features, our dynamic modeling provided important insights into their behavior. At the transcript level, *CASP9* and *CASP3* gene expression showed no statistically significant differences across clusters, although they were numerically higher in Cluster 3 (S1 Fig., panels n and o, in S1 Text). However, our dynamic simulation revealed a clear pattern influenced by post-translational regulation. The model captured a positive feedback loop between activated caspase-9 and caspase-3, which amplified the signal specifically in Cluster 3. This interaction resulted in significantly higher functional activities (Casp9* and Casp3*) in this cluster (S3 Fig. in S1 Text), a distinction that was not observable in the static gene expression data.

### Validating the framework with an independent dataset

To validate the framework, we retrieved independent transcriptomic data (GSE205725) for 16 samples, 10 of which were still disease-free as of the latest record (healthy) and the other six of which had later been diagnosed with breast cancer (susceptible). Before applying the presented workflow to the 16 subjects from the validation dataset, principal component analysis of the transcriptomic data revealed a batch effect between the primary 30 samples from GSE166044 and the validation dataset (GSE205725) (S4 Fig., panel a, in S1 Text). We applied the ComBat function in the R sva package to reduce the batch effect [43] (S4 Fig., panel b, in S1 Text). Then, based on the features extracted from dynamic simulations, the validation dataset samples were assigned to one of the four clusters (derived from the previous 30 samples) by calculating the Euclidean distance of each sample to the cluster centroids and assigning it to the closest cluster. S5 Figure in S1 Text compares model predictions with recorded data. The prediction error, calculated as the difference between the predicted disease-free period based on the median year of the cluster to which each subject was assigned and the actual disease-free period recorded in the database for each subject, is 3.57 years. The higher error rate compared to the previous 30 subjects (2.38 years) likely reflects residual batch effects that persist even after applying the ComBat function to reduce technical differences. Additionally, the small cohort size may restrict the model’s ability to fully capture the complex relationships between signaling behaviors and long-term disease progression.

### Comparing our modeling approach with gene expression-based clustering

To determine whether our modeling approach surpasses using gene expression data alone, we applied the same workflow to gene expression data. The feature importance method based on Random Forest and clustering analysis selected the top 10 genes out of 21,350 genes from the transcriptome data, with *k* = 2, using the Manhattan distance method and the ward.D hierarchical clustering method as the best clustering parameters (S6 Table in S1 Text). The two resulting clusters (S6 Fig. in S1 Text), with median disease-free periods of 10.59 and 7.48, respectively, showed a significant difference between the two groups, but with a relatively high p-value of 0.04 (S7 Fig. in S1 Text). Additionally, stratifying subjects into two clusters provides less detail on the biological heterogeneity of the disease.

Consequently, we further employed the clustering parameters, but with *k* = 4 to examine whether increased biological heterogeneity could be detected. However, this resulted in a loss of statistical significance for the overall model (p-value = 0.1982) (S8 Fig. in S1 Text). Furthermore, the pairwise comparison shows that only the difference between Cluster 1 and Cluster 2 is statistically significant (p-value of 0.019), while the separation between all other cluster pairs is not statistically distinguishable.

We also utilized expression data of genes identified as important components in our dynamic modeling (top 26 features in Fig 4). These genes include *RPS6KA2*, *MAPK1*, *CCNE1*, *FOS*, *TNFSF11*, *CDKN1A*, *E2F1*, *RB1*, *CYCS*, and *DUSP5*. The clustering analysis based on the best parameter set (S7 Table in S1 Text) again identified two clusters with a relatively high p-value of 0.04 (S9 and S10 Figs. in S1 Text). Therefore, using gene expression snapshots without dynamic interactions among key components failed to distinguish biologically relevant sub-groups.

## Discussion

In this study, we explored the dynamic modeling of signaling pathways in breast cancer risk assessment, emphasizing the limitations of static gene expression snapshots. Our findings highlight how integrating personalized transcriptomic data with a mathematical model can provide deeper insights into the complex interactions governing breast cancer development.

Based on personalized models of 30 individuals, our framework stratified the subjects into four clusters with varying degrees of breast cancer susceptibility. Cluster 1 was identified as having the lowest risk for breast cancer development, characterized by a pattern of low pERK/pRSK/c-Fos. Cluster 4 was categorized as the highest risk, with a median disease-free period of 6.05 years. A high pERK/pRSK/c-Fos signal characterizes the signature protein pattern found in Cluster 4. For Clusters 2 and 3, our dynamic model that accounts for the post-translational interaction between pERK and pRSK captured the moderate level of the phosphorylated RSK (pRSK) as a result of high expression of *MAPK1* (encoding ERK) and low expression of *RPS6KA2* (encoding RSK), distinguishing Clusters 2 and 3 from the lowest-risk Cluster 1 and the highest-risk Cluster 4. The risk levels are also indicated by c-Fos protein levels from our personalized dynamic modeling, which could not be inferred from transcriptomic data alone because snapshot *FOS* gene expression levels are not significantly different across the four clusters. Notably, several subjects in Cluster 4, which is linked to high breast cancer risk, had low Gail scores (Fig 5). This suggests that tracking the dynamics of pERK, pRSK, and c-Fos could serve as useful biomarkers for accurately assessing breast cancer risk.

The median disease-free period for Cluster 3 is slightly shorter than that for Cluster 2, although statistical testing did not show a significant difference. We attributed this difference to the higher expression level of *TNFSF11* (which encodes RANKL) in Cluster 3. The elevated RANKL levels stimulate the accumulation of cyclin D, which then post-translationally inactivates RB, allowing E2F1 to be highly active in subjects from Cluster 3. This may explain the higher degree of susceptibility of Cluster 3 compared to Clusters 1 and 2.

Clustering subjects into distinct risk categories based on their signaling pathway simulation profiles highlights the potential for personalized medicine strategies. For example, recent drug discovery efforts that focus on directly inhibiting ERK1/2 to overcome acquired drug resistance and offer more effective cancer treatments [31] may benefit subjects in our Cluster 4. In addition, for subjects in Cluster 3, who exhibited dysregulation of the RB/E2F axis, emerging therapeutic strategies targeting this oncogenic pathway may offer further opportunities for tailored intervention [42].

In conclusion, our work provides a framework for personalized breast cancer risk assessment that integrates dynamic modeling with transcriptomic data. The approach not only effectively predicts the early breast cancer risk but also explains the possible underlying mechanisms of cancer development. Therefore, our approach may aid risk assessment in addition to existing screening tools, such as the Gail model, and support other diagnostic measures. Future studies should aim to validate these findings in larger datasets and explore the clinical applicability of this model in improving breast cancer screening and prevention strategies.

## Materials and methods

### Ethics statement and data access

The current study involves the secondary analysis of existing, de-identified datasets. Whole transcriptome data for the 30 subjects in the primary cohort and 16 subjects in the validation cohort were retrieved from the Gene Expression Omnibus (GEO) database under accession numbers GSE166044 and GSE205725, respectively. Clinical data, including disease-free periods and diagnosis dates, were obtained from the Virtual Tissue Bank (VTB), maintained by the Susan G. Komen Tissue Bank (KTB). The data were accessed on 29 April 2025. All data were accessed in an anonymized format. The authors had no access to information that could identify individual participants during or after data collection. Therefore, this research was exempt from additional institutional review board approval, and informed consent had been obtained from all participants at the time of the original data collection.

### Dynamic modeling

A mathematical model was constructed using ordinary differential equations (ODEs) to simulate the dynamic interactions among core components of the cellular proliferation and apoptosis signaling pathways. The model structure was built by combining two previously published models, one focused on proliferation regulation by Imoto & Okada (2019) [21] and the other on the apoptosis pathway by Legewie et al. (2006) [22]. The model equations are listed in S2 and S3 Tables with parameters listed in S4 Table, in S1 Text. The model was further extended to account for the interactions between the components in the signaling pathways and the 28-day hormonal dynamics. Two hormone dynamics were simulated (S1 Text).

### Transcriptomic datasets

The transcriptome data were obtained from the GEO database. The primary dataset (GSE166044) included samples of histologically normal breast tissue donated by 30 women: 15 healthy controls and 15 women who were later diagnosed with breast cancer (the susceptible group). For model validation, an independent dataset (GSE205725) containing samples from six women susceptible to breast cancer was also used. Since datasets from different sources or processed in various batches often show unwanted technical variation (batch effects), Principal Component Analysis (PCA) was first used to identify these differences. The batch effect was then reduced using the ComBat function within the R sva package (version 3.54.0) [43]. The raw count reads were normalized to TPM (transcripts per million) before being integrated into dynamic modeling.

### Feature extraction from dynamic modeling simulations

To quantify the simulated protein dynamics for each individual, we extracted four characteristics for 14 model variables, resulting in a total of 56 features:

1 The Area Under the Curve (AUC): the cumulative activity of the protein over the 28-day simulation period, calculated using the numpy.trapz function.
2 Maximum activity (max): the highest level reached by the protein variable, calculated using the numpy.max function.
3 Minimum activity (min): the lowest level reached by the protein variable, calculated using the numpy.min function.
4 Rate of activity change (absolute slope): the absolute slope between the max and min values in the dynamic simulation.

After calculating these features, all values were standardized using the scale() function in R before proceeding with the clustering analysis.

### Feature importance analysis

A feature selection step was conducted to determine the most relevant features for the risk assessment. The simulations yielded 56 features (four metrics—AUC, max, min, and absolute slope—for 14 model variables). The Random Forest Classifier was used to rank the features by importance score and identify which dynamic characteristics most effectively distinguished between healthy and susceptible subjects (as shown in Fig 4).

### Clustering analysis

Individual subjects were grouped using a systematic hierarchical clustering approach to identify the optimal stratification of breast cancer risk. The analysis systematically tested various parameter combinations, iterating over the number of clusters (*k* = 2–6), different distance metrics (Binary, Canberra, Euclidean, Manhattan, Maximum, and Minkowski), and hierarchical clustering methods (Average, Centroid, Complete, McQuitty, Median, Single, Ward.D, and Ward.D2).

### Disease-free survival analysis

The disease-free period of each subject was calculated as the time between the initial tissue donation date and the cancer diagnosis date. The data were downloaded from the Virtual Tissue Bank (VTB), which is made available by the Susan G. Komen Tissue Bank (KTB). The disease-free survival curve was estimated using the Nelson-Aalen derived survival function in R (survival version 3.8−3). The performance of each clustering method in stratifying disease-free outcomes was assessed using the Fleming-Harrington (FH) test, a weighted log-rank test comparing differences in disease-free curves (implemented with R FHtest version 1.5.1). We set ρ = 1 and ɣ = 1 to specifically evaluate a medium-term difference between the stratified clusters. Finally, the Silhouette coefficient was used to quantify the quality of each clustering configuration, measuring the degree of cluster separation versus cohesion. The optimal parameter set was selected based on a combination of a statistically significant Fleming-Harrington p-value < 0.05 and the highest Silhouette coefficient.

## Supporting information

S1 Text Supporting information text. (DOCX)
