# Supplementary material for: Integrating dynamic modeling of signaling pathways with subject-specific transcriptomic data to assess breast cancer risk
Source: PLoS One. 2026 Aug 13;21(8):e0355838. doi: 10.1371/journal.pone.0355838 (PMC13472372; doi:10.1371/journal.pone.0355838)
Supplement: Supplementary file 1 [file pone.0355838.s003.pdf]

Response to Reviewers
Integrating dynamic modeling of signaling pathways with subject-specific transcriptomic data to assess breast cancer risk

Black = Comments from the editor or reviewer
Red = Authors' responses
Black highlighted = Changes made to the manuscript

Editor's comment
Thank you for submitting your manuscript to PLOS ONE. After careful consideration, we feel that it has merit but does not fully meet PLOS ONE's publication criteria as it currently stands. Therefore, we invite you to submit a revised version of the manuscript that addresses the points raised during the review process.

We would like to thank the editor and the reviewer for taking the time to provide valuable feedback on our work. We have carefully considered all suggestions and made the necessary revisions to the manuscript. We believe these changes have significantly improved the clarity and quality of our work.

Reviewer's comments
Reviewer #1: This study presents an interesting systems biology approach to breast cancer risk stratification by combining transcriptomic data from normal breast tissue with a mechanistic ODE-based signaling model. Rather than relying only on static gene-expression differences, the authors used each subject's RNA-seq profile to personalize simulations of proliferation and apoptosis signaling over a menstrual-cycle-like period, extracted dynamic features from these simulated trajectories, and used them to identify groups with differing future breast cancer risk. The central idea is that hidden differences in signaling dynamics may reveal susceptibility patterns not apparent from gene expression alone. I like this idea; however, I have a few methodological concerns that I believe the authors could clarify to strengthen the interpretation and reproducibility of the work.

We greatly appreciate the reviewer's constructive and positive feedback, which has been carefully considered in revising the manuscript, as presented below.

Major Comments

1. One methodological point that would benefit from clarification concerns the feature extraction from the simulated ODE trajectories. The manuscript describes "maximum activity" and "minimum activity" as features representing the highest and lowest simulated levels, respectively, yet the methods indicate these were calculated using numpy.argmax and numpy.argmin. As these functions return the index (or time point) at which the maximum/minimum occurs rather than the maximum/minimum value itself, it is unclear whether the intended features were activity magnitudes or timing-based features (e.g., time-to-peak or time-to-nadir). If the latter was intentional, this could indeed be biologically meaningful in the context of dynamic signaling behavior, but the

terminology and interpretation should be revised accordingly. If the former was intended, the implementation may need correction (e.g., using numpy.max/numpy.min). The authors should clarify this point and, if necessary, confirm whether the reported clustering and downstream analyses were affected.

We thank the reviewer for pointing this out. The reviewer is correct that numpy.argmax and numpy.argmin return the index (time points) rather than the maximum/minimum values themselves. We acknowledge that our original explanation in the manuscript was incorrect. However, the features used in our analyses were implemented correctly in the code. In our code implementation, we used the indices returned by numpy.argmax and numpy.argmin to index back into the concentration arrays to retrieve the actual peak and nadir values (i.e., y[max_index] and y[min_index]. We have checked that the approach yields the exact same maximum and minimum values as calling the numpy.max and numpy.min functions directly. Therefore, the downstream clustering and statistical analyses were not affected.

To improve clarity and correct the terminology, we have updated the manuscript text to state that the features were calculated using the numpy.max and numpy.min functions for conceptual simplicity.

These changes have been highlighted in the revised manuscript (Lines 562-565):
2. Maximum activity (max): the highest level reached by the protein variable, calculated using the numpy.max function.
3. Minimum activity (min): the lowest level reached by the protein variable, calculated using the numpy.min function.

2. The manuscript would benefit from a clearer explanation of how the RNA-seq data were used to personalize the ODE model for each subject. It is currently unclear whether gene expression values were used as starting concentrations, to adjust production/degradation rates, or to scale other model parameters. This is important because these choices lead to very different biological interpretations. For example, if a subject has higher MAPK1 RNA expression, does that mean the model starts with more ERK protein, produces ERK faster, or increases ERK signaling activity overall? In addition, some modeled outputs, such as phosphorylated ERK (pERK) and phosphorylated RSK (pRSK), reflect protein activation states that are not directly measured by RNA expression alone. A clearer description of how transcriptomic measurements were translated into these dynamic signaling behaviors would help readers evaluate the biological validity and reproducibility of the model.

We agree that a clear explanation of how the RNA-seq data personalizes the ODE parameters, along with its biological interpretation, is essential for clarity and reproducibility. We have added paragraphs detailing these points to the section 'Integrating gene expression into the dynamic model' in the revised manuscript, highlighted in yellow.

Lines 200-230:

We refer to the model described in S2 and S3 Tables in S1 Text as the generic model of breast tissue proliferation and apoptosis pathways. We personalized the generic model into individualized models by adjusting its parameters based on each individual's gene expression profile. Two groups of parameters were adjusted: 1) parameters representing the total concentration of the proteins and 2) parameters representing the synthesis rate of the proteins. For variables whose activities are controlled at the post-translational level, such as protein modification and stoichiometric inhibition (e.g., ERK, RSK, and RB), individual gene expression levels were used to adjust the total protein amount in the individualized models. For variables whose activity is mainly controlled by synthesis and degradation (e.g., c-Fos and CycD), individual gene expression levels were used to adjust the protein's synthesis rate.

For parameters personalized via synthesis rates, the rate of protein production is scaled proportionally to the individual's gene expression level relative to the healthy population mean. In other words, a subject's protein synthesis rate in the model will be higher or lower depending on whether their individual gene expression is higher or lower than the healthy control average.

For parameters personalized via total concentrations, the overall protein level is scaled proportionally to the individual's gene expression compared to the healthy population average. Essentially, a subject's initial pool (total concentration) of the naive (e.g., unphosphorylated) protein form in the model will be higher or lower depending on whether their gene expression exceeds or falls below the healthy control mean. For instance, a person with elevated *MAPK1* expression begins with a larger baseline pool of unphosphorylated ERK. This expansion enhances the system's maximum signaling capacity. As a result, when stimulated by hormonal oscillations, a person with higher *MAPK1* expression will produce more active, phosphorylated ERK (pERK), thereby speeding up downstream signaling processes (e.g., the conversion of inactive RSK to active pRSK).

These parameter adjustments reflect the underlying biology. While transcript levels inform baseline protein abundance, the actual signaling activity is further shaped by post-translational modifications. Overall, the core concept of our methodology is that transcriptomic data provide a static snapshot of transcript abundance, and the ODE model translates these static inputs into dynamic, post-translationally regulated activation profiles (such as pERK and pRSK) via kinetic interactions and hormone-driven rate equations.

Minor Comments:

1. Lines 82–87 would benefit from clearer wording and additional context. The statement that breast tissue adjacent to tumors displays "unique biological characteristics" is vague—please specify which characteristics are being referenced and provide appropriate supporting citations. In addition, the discussion of normal breast tissue expression subtypes sharing features with the claudin-low phenotype

(lines 86–87) is somewhat confusing and could be clarified to better explain its relevance to breast cancer susceptibility.

We thank the reviewer for the opportunity to clarify this section. We have updated the paragraphs to specify the distinct molecular changes in benign-appearing breast tissue samples adjacent to breast tumors and to explain the relevance of the claudin-low phenotype to cancer susceptibility. The revised paragraphs in the manuscript (highlighted in yellow) are as follows:

Lines 83-115:
Research indicates that gene expression in patient-derived, benign-appearing breast tissue samples adjacent to breast tumors displays distinct and prognostically relevant transcriptomic subtypes [8, 9]. Román-Pérez et al. (2012) first established this by identifying two distinct transcriptomic subtypes, Active and Inactive, in cancer-adjacent benign-appearing tissue. The Active subtype was characterized by elevated expression of genes involved in cellular movement and fibroblast activation, and shared distinct features with the claudin-low breast cancer subtype, notably reduced expression of cell adhesion and cell-cell contact genes. The Active subtype conferred a hazard ratio of approximately 2.5-2.6 for worse overall survival, specifically among ER-positive and hormone-treated patients [8]. This was subsequently validated by Troester et al. (2016) using TCGA data, in which the same two transcriptomic clusters were reproduced in a large multi-institutional cohort, and the Active subtype remained significantly associated with worse 10-year survival among ER-positive patients in multivariate analysis [9].

Critically, subsequent work demonstrated that this risk-associated Active transcriptome phenotype is not restricted to cancer-adjacent tissue but is detectable in histologically normal breast tissue from healthy women. Kang et al. (2020) found that over 50% of breast tissue samples from healthy donors with no history of breast disease displayed the Active transcriptome phenotype [10]. Within this cohort, donors expressing the Active transcriptome phenotype had significantly higher Gail risk scores than those with the Inactive phenotype, providing a direct link between the transcriptomic phenotype and an established clinical risk metric. Notably, most subjects from whom the samples were taken had Gail scores below the clinical eligibility threshold and were not qualified for endocrine prevention therapy, suggesting that transcriptomic profiling may identify at-risk individuals who would otherwise go undetected by conventional risk assessment [10].

In another study, expression profiling of normal breast tissues from healthy women has identified a tissue subtype that shares the same molecular features as the claudin-low breast cancer phenotype, including downregulation of epithelial markers and enrichment of stem cell-like and mesenchymal characteristics [11]. The presence of this stem-like signature in histologically normal tissue suggests a baseline enrichment of cells with high plasticity and dedifferentiation potential, which may be associated with a high susceptibility to oncogenic transformation [11].

Crucially, these findings demonstrate that histologically normal breast tissue may contain early molecular indicators of susceptibility before any clinical or morphological changes, therefore justifying the use of normal tissue transcriptomics to assess and predict future breast cancer risk.

References:

8. Román-Pérez E, Casbas-Hernández P, Pirone JR, Rein J, Carey LA, Lubet RA, et al. Gene expression in extratumoral microenvironment predicts clinical outcome in breast cancer patients. Breast Cancer Res. 2012;14(2):R51. doi: https://doi.org/10.1186/bcr3152.
9. Troester MA, Hoadley KA, D'Arcy M, Cherniack AD, Stewart C, Koboldt DC, et al. DNA defects, epigenetics, and gene expression in cancer-adjacent breast: a study from The Cancer Genome Atlas. npj Breast Cancer. 2016;2(1):16007. doi: https://doi.org/10.1038/npjbcancer.2016.7.
10. Kang T, Yau C, Wong CK, Sanborn JZ, Newton Y, Vaske C, et al. A risk-associated Active transcriptome phenotype expressed by histologically normal human breast tissue and linked to a pro-tumorigenic adipocyte population. Breast Cancer Res. 2020;22(1):81. doi: https://doi.org/10.1186/s13058-020-01322-6.
11. Haakensen VD, Lingjærde OC, Lüders T, Riis M, Prat A, Troester MA, et al. Gene expression profiles of breast biopsies from healthy women identify a group with claudin-low features. BMC Med Genomics. 2011;4(1):77. doi: https://doi.org/10.1186/1755-8794-4-77.

2. For the reported differences in gene expression between susceptible and non-susceptible groups (e.g., MAPK1, RPS6KA2, CCND1, RB1, CDKN1A, SKP2, and CYCS), please specify the statistical test used, along with the corresponding p-values (or adjusted p-values) and effect sizes where applicable. The statement that CYCS was "less statistically significant" is particularly unclear without quantitative reporting.

We thank the reviewer for pointing this out. To address the reviewer's comment, we have performed a Wilcoxon rank-sum test and calculated Cohen's d effect sizes for the comparison between Susceptible and Healthy Control groups across all 15 genes in our model.

We have added the p-values calculated using the Wilcoxon test and adjusted using the Benjamini-Hochberg (FDR) method directly into Figure 2. We have also added S5 Table to the Supporting Information to report all p-values and Cohen's d values.

In the manuscript text (Line 188), we have rephrased the sentence containing "less statistically significant" to "the statistical evidence for the difference was less marked (adjusted p-value of 0.0491)".
