# Supplementary material for: Integrating dynamic modeling of signaling pathways with subject-specific transcriptomic data to assess breast cancer risk
Source: PLoS One. 2026 Aug 13;21(8):e0355838. doi: 10.1371/journal.pone.0355838 (PMC13472372; doi:10.1371/journal.pone.0355838)
Supplement: Supplementary file 2 [file pone.0355838.s001.docx]

**Supporting Information S1 Text**

**Integrating dynamic modeling of signaling pathways with subject-specific transcriptomic data to assess breast cancer risk**

Piyanut Ratphibun Yamashita, Anuwat Tangthanawatsakul, Teerasit Termsaithong, Yaowaluck Maprang Roshorm, Teeraphan Laomettachit

**Periodic functions of ovarian hormone fluctuations**

The fluctuations of hormones estrogen (E2) and progesterone (P4) were modeled using periodic functions based on the equations proposed by Harris (2002) [1].

$[\text{E2}]\left( t \right)= e_{0}+e_{1}e^{-\frac{\left( t-11 \right)^{2}}{e_{2}}}+e_{3}e^{-\frac{\left( t-20 \right)^{2}}{e_{4}}}+e_{1}e^{-\frac{\left( t-39 \right)^{2}}{e_{2}}}$ $+e_{3}e^{-\frac{\left( t-48 \right)^{2}}{e_{4}}}$

Eq. S1

$$\text{[P4]}\left( t \right)= {pr}_{0}+{pr}_{1}e^{-\frac{\left( t-19 \right)^{2}}{{pr}_{2}}}+{pr}_{1}e^{-\frac{\left( t-47 \right)^{2}}{{pr}_{2}}}$$

Eq. S2

**S1 Table.** The parameters in the ovarian hormone fluctuation functions. Parameter values were adapted from Harris (2002) [1] and the time unit was changed to minutes.

| **Parameter** | **Value** | **Unit** |
| --- | --- | --- |
| $e_{0}$ | 62.48 | ng/L |
| $e_{1}$ | 230 | ng/L |
| $e_{2}$ | 5 × 1440^2^ | minute^2^ |
| $e_{3}$ | 115 | ng/L |
| $e_{4}$ | 20 × 1440^2^ | minute^2^ |
| ${pr}_{0}$ | 0.8 | nmol/L |
| ${pr}_{1}$ | 52.24 | nmol/L |
| ${pr}_{2}$ | 19.25 × 1440^2^ | minute^2^ |

**S2 Table.** Equations for the variables in the proliferation pathway.

| Variable description | Equation | Equation number |
| --- | --- | --- |
| Phosphorylated form of ERK | $\frac{\text{d}\left[ \text{pERK} \right]}{\text{d}t}=k_{\text{phos-pERK-E2}}\cdot[\text{E2}]\cdot(\left[ \text{tERK} \right]-\left[ \text{pERK} \right]-[\text{pERK:DUSP}])-k_{\text{dephos-pERK}}\cdot[\text{pERK}]$  $-k_{\text{asso-pERK-DUSP}}\cdot\left[ \text{pERK} \right]\cdot\left( \left[ \text{tDUSP} \right]-\left[ \text{pERK:DUSP} \right] \right)+k_{\text{diss-pERK:DUSP}}\cdot\left[ \text{pERK:DUSP} \right]$ | Eq. S3 |
| Complex between phosphorylated form of ERK and DUSP | $\frac{\text{d}\left[ \text{pERK:DUSP} \right]}{\text{d}t}=k_{\text{asso-pERK-DUSP}}\cdot\left[ \text{pERK} \right]\cdot\left( \left[ \text{tDUSP} \right]-\left[ \text{pERK:DUSP} \right] \right)-k_{\text{diss-pERK:DUSP}}\text{∙}\left[ \text{pERK:DUSP} \right]$ | Eq. S4 |
| Phosphorylated form of RSK | $\frac{\text{d}\left[ \text{pRSK} \right]}{\text{d}t}=\frac{k_{\text{phos-RSK-pERK}}\cdot\left[ \text{pERK} \right]\cdot(\left[ \text{tRSK} \right]-[\text{pRSK}])}{K_{\text{phos-RSK-pERK}}+\left[ \text{tRSK} \right]-[\text{pRSK}]}-k_{\text{dephos-pRSK}}\cdot[\text{pRSK}]$ | Eq. S5 |
| c-Fos | $\frac{\text{d}\left[ \text{c-}\text{Fos} \right]}{\text{d}t}=\frac{k_{\text{syn-cFos}}\cdot\left[ \text{pERK} \right]\cdot\left[ \text{pRSK} \right]}{K_{\text{syn-cFos}}+\left[ \text{pRSK} \right]}-k_{\text{deg-cFos}}\cdot[\text{c}\text{-}\text{Fos}]$ | Eq. S6 |
| Cyclin D | $\frac{\text{d}\left[ \text{CycD} \right]}{\text{d}t}=k_{\text{syn-CycD-cFos}}\cdot[\text{c}\text{-}\text{Fos}]+\frac{k_{\text{syn-CycD-RANKL}}\cdot\left[ \text{RANKL} \right]}{K_{\text{syn-CycD-RANKL}}+\left[ \text{RANKL} \right]}-k_{\text{deg-CycD}}\cdot[\text{CycD}]$ | Eq. S7 |
| Unphosphorylated form of RB (both free form and complexed with E2F) | $\frac{\text{d}\left[ \text{uRB} \right]}{\text{d}t}=-k_{\text{phos-uRB-CycD}}\cdot\left[ \text{CycD} \right]\cdot\left[ \text{uRB} \right]-k_{\text{phos-uRB-CycE}}\cdot(\left[ \text{tCycE} \right]-\left[ \text{CycE:p21} \right])\cdot\left[ \text{uRB} \right]+k_{\text{dephos-pRB}}\cdot\left( \left[ \text{tRB} \right]-\left[ \text{uRB} \right] \right)$ | Eq. S8 |
| Total E2F (both free form and complexed with RB) | $\frac{\text{d}\left[ \text{tE2F} \right]}{\text{d}t}=k_{\text{syn-E2F}}+\frac{k_{\text{syn-E2F-E2F}}\cdot[\text{E2F}]}{K_{\text{syn-E2F-E2F}}+[\text{E2F}]}-k_{\text{deg-E2F}}\cdot[\text{tE2F}]$ | Eq. S9 |
| Free E2F | $\frac{\text{d}\left[ \text{E2F} \right]}{\text{d}t}=k_{\text{syn-E2F}}+\frac{k_{\text{syn-E2F-E2F}}\cdot\left[ \text{E2F} \right]}{K_{\text{syn-E2F-E2F}}+\left[ \text{E2F} \right]}-k_{\text{deg-E2F}}\cdot\left[ \text{E2F} \right]$ $+k_{\text{phos-uRB-CycD}}\cdot\left[ \text{CycD} \right]\cdot\left( \left[ \text{tE2F} \right]-\left[ \text{E2F} \right] \right)$ $+k_{\text{phos-uRB-CycE}}\cdot\left( \left[ \text{tCycE} \right]-\left[ \text{CycE:p21} \right] \right)\cdot\left( \left[ \text{tE2F} \right]-\left[ \text{E2F} \right] \right)$ $-k_{\text{asso-E2F-uRB}}\cdot\left[ \text{E2F} \right]\cdot\left( \left[ \text{uRB} \right]-\left[ \text{tE2F} \right]+\left[ \text{E2F} \right] \right)$ $+k_{\text{diss-E2F:uRB}}\cdot(\left[ \text{tE2F}]-[\text{E2F} \right])$ | Eq. S10 |
| Total cyclin E (both free form and complexed with p21) | $\frac{\text{d}\left[ \text{tCycE} \right]}{\text{d}t}=k_{\text{syn-CycE-E2F}}\cdot\left[ \text{E2F} \right]-k_{\text{deg-CycE}}\cdot[\text{tCycE}]$ | Eq. S11 |
| Complex between cyclin E and p21 | $\frac{\text{d}\left[ \text{CycE:p21} \right]}{\text{d}t}=k_{\text{asso-CycE-p21}}\cdot\left( \left[ \text{tCycE} \right]-\left[ \text{CycE:p21} \right] \right)\cdot\left( \left[ \text{tp21} \right]-\left[ \text{CycE:p21} \right] \right)$ $-k_{\text{diss-CycE:p21}}\cdot\left[ \text{CycE:p21} \right]$ $-k_{\text{deg-CycE}}\cdot\left[ \text{CycE:p21} \right]$ $-(k_{\text{dephos-p21}}+k_{\text{dephos-p21-cy}}\cdot[\text{SKP2}]\cdot(\left[ \text{tCycE} \right]-[\text{CycE:p21}])+[\text{pAKT]})\cdot[\text{CycE:p21}]$ | Eq. S12 |
| RANKL | $\frac{\text{d}\left[ \text{RANKL} \right]}{\text{d}t}=\frac{k_{\text{syn-RANKL}}\cdot\left[ \text{P4} \right]}{K_{\text{syn-RANKL}}+\left[ \text{P4} \right]}-k_{\text{deg-}\text{RANKL}}\cdot[\text{RANKL}]$ | Eq. S13 |

**S3 Table.** Equations for the variables in the apoptosis pathway.

| Variable description | Equation | Equation number |
| --- | --- | --- |
| Apoptotic protease activating factor-1 (Apaf-1) (We assume Apaf-1 concentration equals cytochrome c.) | $\frac{\text{d}\left[ \text{CytoC} \right]}{\text{d}t}=\frac{k_{\text{act-CytoC-E2}}\cdot[\text{E2]}}{K_{\text{act-CytoC-E2}}+\left[ \text{E2} \right]}-k_{\text{deact-CytoC}}\cdot\left[ \text{CytoC} \right]$ $-k_{\text{asso-CytoC-C9}}\cdot\left[ \text{CytoC} \right]\cdot\left[ \text{Casp9} \right]+k_{\text{diss-CytoC:C9}}\cdot\left[ \text{CytoC:Casp9} \right]$ $-k_{\text{asso-CytoC-C9*}}\cdot\left[ \text{CytoC} \right]\cdot\left[ \text{Casp9*} \right]+k_{\text{diss-CytoC:C9*}}\cdot\left[ \text{CytoC:Casp9*} \right]$ $-k_{\text{asso-CytoC-C9:X}}\cdot\left[ \text{CytoC} \right]\cdot\left[ \text{Casp9:X} \right]+k_{\text{diss-CytoC:C9:X}}\cdot\left[ \text{CytoC:Casp9:X} \right]$ $-k_{\text{asso-CytoC-C9*:X}}\cdot\left[ \text{CytoC} \right]\cdot\left[ \text{Casp9*:X} \right]+k_{\text{diss-CytoC:C9*:X}}\cdot\left[ \text{CytoC:Casp9*:X} \right]$ | Eq. S14 |
| Free procaspase-9 | $\frac{\text{d}\left[ \text{C}\text{asp}\text{9} \right]}{\text{d}t}=k_{\text{syn-C9}}-k_{\text{deg}}\cdot\left[ \text{C}\text{asp}\text{9} \right]$ $-k_{\text{asso-CytoC-C9}}\cdot\left[ \text{CytoC} \right]\cdot\left[ \text{C}\text{asp}\text{9} \right]+k_{\text{diss-CytoC:C9}}\cdot\left[ \text{CytoC}\text{:C}\text{asp}\text{9} \right]$ $-k_{\text{asso-C9-X}}\cdot\left[ \text{C}\text{asp}\text{9} \right]\cdot\left[ \text{X} \right]+k_{\text{diss-C9:X}}\cdot\left[ \text{C}\text{asp}\text{9:X} \right]$ $-k_{\text{act-C9-C3*}}\cdot\left[ \text{C}\text{asp}\text{9} \right]\cdot\left[ {\text{C}\text{asp}\text{3}}^{\text{*}} \right]$ | Eq. S15 |
| Complex between procaspase-9 and XIAP | $\frac{\text{d}\left[ \text{C}\text{asp}\text{9:X} \right]}{\text{d}t}=k_{\text{asso-C9-X}}\cdot[\text{C}\text{asp}\text{9}]\cdot[\text{X}]-k_{\text{diss-C9:X}}\cdot[\text{C}\text{asp}\text{9:X}]$ $-k_{\text{asso-}\text{CytoC-C9:X}}\cdot\text{[}\text{CytoC}\text{]}\cdot\text{[}\text{C}\text{asp}\text{9:X]}+k_{\text{diss-CytoC:C9:X}}\cdot\text{[CytoC:C}\text{asp}\text{9:X}]$ $-k_{\text{deg}}\cdot\text{[C}\text{asp}\text{9:}\text{X]}$ | Eq. S16 |
| Free XIAP | $\frac{\text{d}\left[ \text{X} \right]}{\text{d}t}=k_{\text{syn-X}}-k_{\text{deg}}\cdot\left[ \text{X} \right]$ $-k_{\text{asso-C9-X}}\cdot\left[ \text{C}\text{asp}\text{9} \right]\cdot\left[ \text{X} \right]+k_{\text{diss-C9:X}}\cdot\left[ \text{C}\text{asp}\text{9:X} \right]$ $-k_{\text{asso-CytoC:C9-X}}\cdot[\text{CytoC}\text{:C}\text{asp}\text{9}]\cdot[\text{X}]+k_{\text{diss}\text{2}\text{-Cyto}\text{C}\text{:C9:X}}\cdot[\text{CytoC}\text{:C}\text{asp}\text{9:X}]$ $-k_{\text{asso-C3*-X}}\cdot\text{[C}\text{asp}\text{3*}\text{]}\cdot[\text{X}]+k_{\text{diss-C3*:X}}\cdot[\text{C}\text{asp}\text{3*:X}]$ $-k_{\text{asso-C9*-X}}\cdot\text{[C}\text{asp}\text{9*] }\text{∙ [}\text{X]}+k_{\text{diss-C9*:X}}\cdot\text{[C}\text{asp}\text{9*:X]}$ $-k_{\text{asso-CytoC:C9*-X}}\cdot\text{[CytoC:C}\text{asp}\text{9*}]\cdot[\text{X}]+k_{\text{diss}\text{2}\text{-Cyto}\text{C}\text{:C9*:X}}\cdot\text{[CytoC:C}\text{asp}\text{9*:X]}$ | Eq. S17 |
| Complex between Apaf-1, procaspase-9, and XIAP | $\frac{\text{d}\left[ \text{CytoC:C}\text{asp}\text{9:X} \right]}{\text{d}t}=k_{\text{asso-CytoC:C9-X}}\cdot[\text{CytoC:Casp9}]\cdot[\text{X}]-k_{\text{diss}\text{2}\text{-Cyto}\text{C}\text{:C9:X}}\cdot[\text{CytoC:Casp9:X}]$ $+k_{\text{asso-CytoC-C9:X}}\cdot\text{[}\text{CytoC}]\cdot[\text{Casp9:X]}-k_{\text{diss-CytoC:C9:X}}\cdot[\text{CytoC:Casp9:X}]$ $-k_{\text{deg}}\cdot[\text{CytoC:Casp9:X}]$ | Eq. S18 |
| Complex between Apaf-1 and procaspase-9 | $\frac{\text{d}\left[ \text{CytoC:C}\text{asp}\text{9} \right]}{\text{d}t}=k_{\text{asso-CytoC-C9}}\cdot[\text{CytoC}]\cdot[\text{Casp9}]-k_{\text{diss-CytoC:C9}}\cdot[\text{CytoC}\text{:Casp9}]$ $-k_{\text{asso-CytoC:C9-X}}\cdot[\text{CytoC}\text{:Casp9}]\cdot[\text{X}]+k_{\text{diss}\text{2}\text{-Cyto}\text{C}\text{:C9:X}}\cdot[\text{CytoC}\text{:Casp9:X}\text{]}$ $-k_{\text{act-CytoC:C9-C3*}}\cdot\text{[}\text{CytoC}\text{:Casp9}]\cdot[\text{Casp3*]}\text{}$ ${-k}_{\text{deg}}\cdot\text{[}\text{CytoC}\text{:Casp9]}\text{ }$ | Eq. S19 |
| Free procaspase-3 | $\frac{\text{d}\left[ \text{C}\text{asp}\text{3} \right]}{\text{d}t}=k_{\text{syn-C3}}-k_{\text{deg}}\cdot\left[ \text{C}\text{asp}\text{3} \right]$ ${-k}_{\text{act-C3-C9}}\cdot\text{[}\text{Casp3]}\cdot\text{[}\text{Casp9]}\text{}$ $-k_{\text{act-C3-CytoC:C9}}\cdot\text{[}\text{Casp3]}\cdot\text{[CytoC}\text{:Casp9]}\text{}$ $-k_{\text{act-C3-C9*}}\cdot\text{[}\text{C}\text{asp}\text{3]}\cdot\text{[C}\text{asp}\text{9*]}$ $-k_{\text{act-C3-CytoC:C9*}}\cdot\text{[}\text{C}\text{asp}\text{3]}\cdot\text{[}\text{CytoC:C}\text{asp}\text{9*}\text{]}$ | Eq. S20 |
| Activated caspase-3 | $\frac{\text{d}\left[ \text{C}\text{asp}\text{3*} \right]}{\text{d}t}=k_{\text{act-C3-C9}}\cdot\text{[}\text{C}\text{asp}\text{3]}\cdot\text{[}\text{C}\text{asp}\text{9]}\text{}$ $+k_{\text{act-C3-CytoC:C9}}\cdot\text{[}\text{C}\text{asp}\text{3]}\cdot\text{[CytoC}\text{:C}\text{asp}\text{9]}\text{}$ $+k_{\text{act-C3-C9*}}\cdot\text{[}\text{C}\text{asp}\text{3]}\cdot\text{[C}\text{asp}\text{9*]}$ $+k_{\text{act-C3-CytoC:C9*}}\cdot\text{[}\text{C}\text{asp}\text{3]}\cdot\text{[}\text{CytoC:C}\text{asp}\text{9*}\text{]}\text{}$ $\text{ }{-k}_{\text{asso-C3*-X}}\cdot\text{[}\text{C}\text{asp}\text{3*]}\cdot[\text{X}]+k_{\text{diss-C3*:X}}\cdot[\text{C}\text{asp}\text{3*}\text{:X}]$ $-\text{k}_{\text{deg}}\cdot\text{[}\text{C}\text{asp}\text{3*}\text{]}\text{}$ $-\frac{k_{\text{off-C3*-P4}}\cdot[\text{P4}]\cdot[\text{C}\text{asp}\text{3*}]}{K_{\text{off-C3*-P4}}+[\text{C}\text{asp}\text{3*}]}$ | Eq. S21 |
| Complex between activated caspase-3 and XIAP | $\frac{\text{d}\left[ \text{C}\text{asp}\text{3*:X} \right]}{\text{d}t}=\text{ }k_{\text{asso-C3*-X}}\cdot\text{[}\text{C}\text{asp}\text{3*]}\cdot\left[ \text{X} \right]-k_{\text{diss-C3*:X}}\cdot\left[ \text{C}\text{asp}\text{3*}\text{:X} \right]$ $-\text{k}_{\text{deg}}\cdot\text{[}\text{C}\text{asp}\text{3*:X}\text{]}$ | Eq. S22 |
| Complex between activated caspase-9 and XIAP | $\frac{\text{d}\left[ \text{C}\text{asp}\text{9*}:\text{X} \right]}{\text{d}t}=k_{\text{asso-C9*-X}}\cdot\text{[C}\text{asp}\text{9*] }\text{∙ [}\text{X]}-k_{\text{diss-C9*:X}}\cdot\text{[C}\text{asp}\text{9*:X]}\text{}$ $-k_{\text{asso-CytoC-C9*:X}}\cdot[\text{CytoC}]\cdot\text{[C}\text{asp}\text{9*:X}]+k_{\text{diss-CytoC:C9*:X}}\cdot[\text{CytoC:C}\text{asp}\text{9*:X}]\text{ }\text{}$ $-\text{k}_{\text{deg}}\cdot\text{[}\text{C}\text{asp}\text{9*:X}\text{]}$ | Eq. S23 |
| Activated caspase-9 | $\frac{\text{d}\left[ \text{C}\text{asp}\text{9*} \right]}{\text{d}t}=k_{\text{act-C9-C3*}}\cdot\left[ \text{C}\text{asp}\text{9} \right]\cdot\left[ {\text{C}\text{asp}\text{3}}^{\text{*}} \right]$ $-k_{\text{asso-CytoC-C9*}}\cdot\left[ \text{CytoC} \right]\cdot\left[ \text{Casp9*} \right]+k_{\text{diss-CytoC:C9*}}\cdot\left[ \text{CytoC:Casp9*} \right]$ $-k_{\text{asso-C9*-X}}\cdot\text{[C}\text{asp}\text{9*] }\text{∙ [}\text{X]}+k_{\text{diss-C9*:X}}\cdot\text{[C}\text{asp}\text{9*:X]}\text{}$ $-\text{k}_{\text{deg}}\cdot\text{[}\text{C}\text{asp}\text{9*}\text{]}$ | Eq. S24 |
| Complex between Apaf-1 and activated caspase-9 | $\frac{\text{d}\left[ \text{CytoC:C}\text{asp}\text{9*} \right]}{\text{d}t}=k_{\text{act-CytoC:C9-C3*}}\cdot\text{[}\text{CytoC}\text{:Casp9}]\cdot[\text{Casp3*]}\text{}$ $+k_{\text{asso-CytoC-C9*}}\cdot\left[ \text{CytoC} \right]\cdot\left[ \text{Casp9*} \right]-k_{\text{diss-CytoC:C9*}}\cdot\left[ \text{CytoC:Casp9*} \right]$ $-k_{\text{asso-CytoC:C9*-X}}\cdot\text{[CytoC:Casp9*}]\cdot[\text{X}]+k_{\text{diss}\text{2}\text{-Cyto}\text{C}\text{:C9*:X}}\cdot\text{[CytoC:Casp9*:X] }\text{}$ $-\text{k}_{\text{deg}}\cdot\text{[CytoC:}\text{C}\text{asp}\text{9*}\text{]}$ | Eq. S25 |
| Complex between Apaf-1 and activated caspase-9, and XIAP | $\frac{\text{d}\left[ \text{CytoC:C}\text{asp}\text{9*:X} \right]}{\text{d}t}=k_{\text{asso-CytoC:C9*-X}}\cdot\text{[CytoC:Casp9*}]\cdot[\text{X}]-k_{\text{diss}\text{2}\text{-Cyto}\text{C}\text{:C9*:X}}\cdot\text{[CytoC:Casp9*:X] }\text{}$ $+k_{\text{asso-CytoC-C9*:X}}\cdot\left[ \text{CytoC} \right]\cdot\left[ \text{Casp9*:X} \right]-k_{\text{diss-CytoC:C9*:X}}\cdot\left[ \text{CytoC:Casp9*:X} \right]\text{ }\text{}$ $-\text{k}_{\text{deg}}\cdot\text{[CytoC:}\text{C}\text{asp}\text{9*:X}\text{]}$ | Eq. S26 |

**S4 Table.** Model parameters.

| **Parameter** | **Value** | **Reference** |
| --- | --- | --- |
| $k_{\text{phos-pERK-E2}}$ | 0.004070 nM^−1^ min^−1^ | Estimated from Scaling et al. [2] |
| $k_{\text{dephos-pERK}}$ | 0.09564 min^−1^ | Value from Imoto & Okada [3] |
| $k_{\text{asso-pERK-DUSP}}$ | 0.01 nM^−1^ min^−1^ |  |
| $k_{\text{diss-pERK:DUSP}}$ | 0.002281 min^−1^ |  |
| $k_{\text{phos-RSK-pERK}}$ | 1.735 min^−1^ |  |
| $K_{\text{phos-RSK-pERK}}$ | 99.36 nM |  |
| $k_{\text{dephos-pRSK}}$ | 0.9034 min^−1^ |  |
| $k_{\text{syn-cFos}}$ | 0.006149 min^−1^ |  |
| $K_{\text{syn-cFos}}$ | 0.1123 nM |  |
| $k_{\text{deg-cFos}}$ | 0.01475 min^−1^ |  |
| $k_{\text{syn-CycD-cFos}}$ | 0.0045 min^−1^ |  |
| $k_{\text{syn-CycD-RANKL}}$ | 16.8 nM min^−1^ | Estimated from Tanos et al. [4] |
| $K_{\text{syn-CycD-RANKL}}$ | 7.44162 nM |  |
| $k_{\text{deg-CycD}}$ | 0.069 min^−1^ | Value adjusted from Imoto & Okada [3] |
| $k_{\text{phos-uRB-CycD}}$ | 0.2 nM^−1^ min^−1^ | Value from Imoto & Okada [3] |
| $k_{\text{phos-uRB-CycE}}$ | 0.3 nM^−1^ min^−1^ |  |
| $k_{\text{dephos-pRB}}$ | 0.05 min^−1^ |  |
| $k_{\text{syn-E2F}}$ | 0.03 nM min^−1^ |  |
| $k_{\text{syn-E2F-E2F}}$ | 0.04 nM min^−1^ |  |
| $K_{\text{syn-E2F-E2F}}$ | 0.2 nM |  |
| $k_{\text{deg-E2F}}$ | 0.05 min^−1^ |  |
| $k_{\text{asso-E2F-uRB}}$ | 5.0 nM^−1^ min^−1^ |  |
| $k_{\text{diss-E2F:uRB}}$ | 0.005 min^−1^ |  |
| $k_{\text{syn-CycE}\text{-E2F}}$ | 0.01 min^−1^ |  |
| $k_{\text{deg-CycE}}$ | 0.004 min^−1^ |  |
| $k_{\text{asso-CycE-p21}}$ | 1.0 nM^−1^ min^−1^ |  |
| $k_{\text{diss-CycE:p21}}$ | 0.05 min^−1^ |  |
| $k_{\text{dephos}\text{-}\text{p21}}$ | 0.0025 min^−1^ |  |
| $k_{\text{dephos}\text{-}\text{p21}\text{-}\text{cy}}$ | 0.007 nM^−2^ min^−1^ |  |
| $k_{\text{syn-RANKL}}$ | 0.39 nM min^−1^ | Estimated from Tanos et al. [4] |
| $K_{\text{syn-RANKL}}$ | 163.09392 nM |  |
| $k_{\text{deg-RANKL}}$ | 0.06 min^−1^ | Estimated |
| $k_{\text{act-CytoC-E2}}$ | 0.011048 nM min^−1^ | Estimated from Deng et al. [5] |
| $K_{\text{act-CytoC-E2}}$ | 4.36 nM |  |
| $k_{\text{deact-CytoC}}$ | 0.006 min^−1^ | Estimated |
| $k_{\text{asso-CytoC-C9}}$ | 0.12 nM^−1^ min^−1^ | Value from Legewie et al. [6] |
| $k_{\text{diss-CytoC:C9}}$ | 6.0 min^−1^ |  |
| $k_{\text{asso-CytoC-C9*}}$ | 0.12 nM^−1^ min^−1^ |  |
| $k_{\text{diss-CytoC:C9*}}$ | 6.0 min^−1^ |  |
| $k_{\text{asso-CytoC-C9:X}}$ | 0.12 nM^−1^ min^−1^ |  |
| $k_{\text{diss-CytoC:C9:X}}$ | 6.0 min^−1^ |  |
| $k_{\text{asso-CytoC-C9*:X}}$ | 0.12 nM^−1^ min^−1^ |  |
| $k_{\text{diss-CytoC:C9*:X}}$ | 6.0 min^−1^ |  |
| $k_{\text{syn-C9}}$ | 1.2 nM min^−1^ |  |
| $k_{\text{deg}}$ | 0.06 min^−1^ |  |
| $k_{\text{asso-C9-X}}$ | 0.06 nM^−1^ min^−1^ |  |
| $k_{\text{diss-C9:X}}$ | 0.06 min^−1^ |  |
| $k_{\text{act-C9-C3*}}$ | 0.012 nM^−1^ min^−1^ |  |
| $k_{\text{syn-X}}$ | 2.4 nM min^−1^ |  |
| $k_{\text{asso-CytoC:C9-X}}$ | 0.06 nM^−1^ min^−1^ |  |
| $k_{\text{diss}\text{2}\text{-Cyto}\text{C}\text{:C9:X}}$ | 0.06 min^−1^ |  |
| $k_{\text{asso-C3*-X}}$ | 0.18 nM^−1^ min^−1^ |  |
| $k_{\text{diss-C3*:X}}$ | 0.06 min^−1^ |  |
| $k_{\text{asso-C9*-X}}$ | 0.06 nM^−1^ min^−1^ |  |
| $k_{\text{diss-C9*:X}}$ | 0.06 min^−1^ |  |
| $k_{\text{asso-CytoC:C9*-X}}$ | 0.06 nM^−1^ min^−1^ |  |
| $k_{\text{diss}\text{2}\text{-CytoC:C9*:X}}$ | 0.06 min^−1^ |  |
| $k_{\text{act-CytoC:C9-C3*}}$ | 0.012 nM^−1^ min^−1^ |  |
| $k_{\text{syn-C3}}$ | 12.0 nM min^−1^ |  |
| $k_{\text{act-C3-C9*}}$ | 0.003 nM^−1^ min^−1^ |  |
| $k_{\text{act-C3-CytoC:C9*}}$ | 0.21 nM^−1^ min^−1^ |  |
| $k_{\text{act-C3-C9}}$ | 0.0003 nM^−1^ min^−1^ |  |
| $k_{\text{act-C3-CytoC:C9}}$ | 0.021 nM^−1^ min^−1^ |  |
| $k_{\text{off-C3*-P4}}$ | 0.2769 min^−1^ | Estimated from Behera et al. [7] |
| $K_{\text{off-C3*-P4}}$ | 4.447583 nM |  |

**S5 Table.** Wilcoxon test results and Cohen’s d values for gene expression levels in Susceptible versus Healthy subjects.

| **Gene** | **p** | **p.adj** | **p.adj.signif** | **effect size (d)** |
| --- | --- | --- | --- | --- |
| *MAPK1* | 0.00002 | 0.00018 | *** | 1.78 |
| *DUSP5* | 0.81300 | 0.81300 | ns | 0.54 |
| *RPS6KA2* | 0.00014 | 0.00070 | *** | 1.73 |
| *FOS* | 0.61400 | 0.65786 | ns | 0.27 |
| *CCND1* | 0.00494 | 0.01852 | * | 1.13 |
| *RB1* | 0.00000 | 0.00002 | **** | 2.37 |
| *E2F1* | 0.43600 | 0.50308 | ns | −0.57 |
| *CCNE1* | 0.06900 | 0.12938 | ns | −0.72 |
| *CDKN1A* | 0.00911 | 0.02278 | * | 1.04 |
| *SKP2* | 0.00674 | 0.02022 | * | 1.09 |
| *AKT1* | 0.23300 | 0.29125 | ns | 0.49 |
| *TNFSF11* | 0.08200 | 0.13667 | ns | −0.54 |
| *CYCS* | 0.02290 | 0.04907 | * | 0.96 |
| *CASP9* | 0.18700 | 0.25500 | ns | −0.42 |
| *CASP3* | 0.17000 | 0.25500 | ns | 0.39 |

p-values were adjusted using the Benjamini-Hochberg (FDR) method. Significance levels: p.adj ≤ 0.0001 (****), p.adj ≤ 0.001 (***), p.adj ≤ 0.01 (**), and p.adj ≤ 0.05 (*). ns: not significant. Cohen's d was calculated to estimate effect sizes (∣d∣ ≥ 0.8 indicates a large effect).

**S1 Figure.** Boxplots of gene expression in the TPM values from the GSE166044 dataset. The data are the same as in Figure 2, but the 30 subjects were grouped into four clusters based on the results of the proposed framework. Bars labeled with different letters are significantly different from one another, as assessed by Tukey’s HSD test after a one-way ANOVA (p < 0.05).

**S2 Figure.** Boxplots of feature values extracted from dynamic modeling simulations: AUC, max, min, and absolute slope for each cluster. The 30 subjects were grouped into four clusters based on the results of the proposed framework. Bars labeled with different letters are significantly different, as determined by Tukey’s HSD test following a one-way ANOVA (p < 0.05).

**S3 Figure.** Dynamic simulations of 30 individual subjects stratified into four clusters based on the results of the proposed framework. (a-e) Cytochrome c (f-j) Casp9* (k-o) Casp3*. Bars labeled with different letters are significantly different from one another, as assessed by Tukey’s HSD test after a one-way ANOVA (p-value < 0.05).

**S4 Figure.** Principal component analysis of the 30 donors (GSE166044) and the 16 donors (validation data) (GSE205725). PCA was performed on the original raw count gene expression data before batch correction (a) and after batch correction using ComBat (b).

**S5 Figure.** Comparison of the median disease-free periods estimated by the Nelson-Aalen derived survival function for the four clusters of the 30 primary subjects and the actual time to breast cancer diagnosis from the validation dataset. (a) Six donors who were later diagnosed with breast cancer (susceptible group). (b) Ten donors who remained breast cancer-free as of the latest record (healthy group).

**S6 Table.** Results of clustering analysis based on 21,350 gene expression profiles. Parameter set 1, highlighted in red, was chosen for further analysis.

| Parameter set | Number of top features used | Number of clusters (*k*) | Distance method | Hierarchical clustering method | p-value | Median disease-free year for each cluster | Number of subjects in each cluster | Silhouette coefficient |
| --- | --- | --- | --- | --- | --- | --- | --- | --- |
| 1 | 10 | 2 | manhattan | ward.D | 0.042 | 10.59, 7.48 | 15, 15 | 0.474 |
| 2 | 10 | 2 | manhattan | complete | 0.042 | 10.59, 7.48 | 15, 15 | 0.474 |
| 3 | 10 | 2 | manhattan | average | 0.042 | 10.59, 7.48 | 15, 15 | 0.474 |
| 4 | 10 | 2 | manhattan | median | 0.042 | 10.59, 7.48 | 15, 15 | 0.474 |
| 5 | 10 | 2 | manhattan | ward.D2 | 0.042 | 10.59, 7.48 | 15, 15 | 0.474 |
| 6 | 11 | 2 | manhattan | ward.D | 0.042 | 10.59, 7.48 | 15, 15 | 0.469 |
| 7 | 11 | 2 | manhattan | complete | 0.042 | 10.59, 7.48 | 15, 15 | 0.469 |
| 8 | 11 | 2 | manhattan | average | 0.042 | 10.59, 7.48 | 15, 15 | 0.469 |
| 9 | 11 | 2 | manhattan | ward.D2 | 0.042 | 10.59, 7.48 | 15, 15 | 0.469 |
| 59 | 26 | 2 | manhattan | ward.D | 0.042 | 10.59, 7.48 | 15, 15 | 0.436 |
| 60 | 26 | 2 | manhattan | mcquitty | 0.042 | 10.59, 7.48 | 15, 15 | 0.436 |
| 61 | 26 | 2 | manhattan | ward.D2 | 0.042 | 10.59, 7.48 | 15, 15 | 0.436 |
| 167 | 26 | 2 | euclidean | ward.D | 0.041 | 10.59, 6.765 | 16, 14 | 0.415 |
| 168 | 26 | 2 | euclidean | complete | 0.041 | 10.59, 6.765 | 16, 14 | 0.415 |
| 169 | 26 | 2 | euclidean | average | 0.041 | 10.59, 6.765 | 16, 14 | 0.415 |
| 170 | 26 | 2 | euclidean | mcquitty | 0.041 | 10.59, 6.765 | 16, 14 | 0.415 |
| 171 | 26 | 2 | euclidean | ward.D2 | 0.041 | 10.59, 6.765 | 16, 14 | 0.415 |
| 172 | 26 | 2 | minkowski | ward.D | 0.041 | 10.59, 6.765 | 16, 14 | 0.415 |
| 173 | 26 | 2 | minkowski | complete | 0.041 | 10.59, 6.765 | 16, 14 | 0.415 |
| 174 | 26 | 2 | minkowski | average | 0.041 | 10.59, 6.765 | 16, 14 | 0.415 |
| 175 | 26 | 2 | minkowski | mcquitty | 0.041 | 10.59, 6.765 | 16, 14 | 0.415 |
| 176 | 26 | 2 | minkowski | ward.D2 | 0.041 | 10.59, 6.765 | 16, 14 | 0.415 |
| 275 | 26 | 2 | canberra | single | 0.041 | 10.59, 6.765 | 16, 14 | 0.389 |
| 276 | 26 | 2 | canberra | complete | 0.041 | 10.59, 6.765 | 16, 14 | 0.389 |
| 342 | 26 | 2 | canberra | ward.D | 0.042 | 10.59, 7.48 | 15, 15 | 0.373 |
| 343 | 26 | 2 | canberra | ward.D2 | 0.042 | 10.59, 7.48 | 15, 15 | 0.373 |
| 422 | 26 | 3 | manhattan | ward.D | 0.039 | 10.59, 6.05, 7.48 | 15, 8, 7 | 0.311 |
| 423 | 26 | 3 | manhattan | ward.D2 | 0.039 | 10.59, 6.05, 7.48 | 15, 8, 7 | 0.311 |
| 437 | 26 | 3 | euclidean | ward.D | 0.042 | 10.59, 6.05, 7.48 | 16, 7, 7 | 0.294 |
| 438 | 26 | 3 | euclidean | ward.D2 | 0.042 | 10.59, 6.05, 7.48 | 16, 7, 7 | 0.294 |
| 439 | 26 | 3 | minkowski | ward.D | 0.042 | 10.59, 6.05, 7.48 | 16, 7, 7 | 0.294 |
| 440 | 26 | 3 | minkowski | ward.D2 | 0.042 | 10.59, 6.05, 7.48 | 16, 7, 7 | 0.294 |
| 457 | 26 | 2 | maximum | ward.D | 0.041 | 10.59, 6.765 | 16, 14 | 0.292 |
| 458 | 26 | 2 | maximum | complete | 0.041 | 10.59, 6.765 | 16, 14 | 0.292 |
| 459 | 26 | 2 | maximum | average | 0.041 | 10.59, 6.765 | 16, 14 | 0.292 |
| 460 | 26 | 2 | maximum | mcquitty | 0.041 | 10.59, 6.765 | 16, 14 | 0.292 |
| 461 | 26 | 2 | maximum | median | 0.041 | 10.59, 6.765 | 16, 14 | 0.292 |
| 462 | 26 | 2 | maximum | ward.D2 | 0.041 | 10.59, 6.765 | 16, 14 | 0.292 |
| 521 | 22 | 2 | maximum | median | 0.036 | 10.59, 6.05 | 17, 13 | 0.278 |
| 522 | 27 | 3 | maximum | mcquitty | 0.026 | 10.59, 7.92, 4.93 | 16, 9, 5 | 0.241 |
| 523 | 26 | 3 | maximum | mcquitty | 0.026 | 10.59, 7.92, 4.93 | 16, 9, 5 | 0.240 |
| 524 | 25 | 3 | maximum | mcquitty | 0.026 | 10.59, 7.92, 4.93 | 16, 9, 5 | 0.237 |
| 525 | 28 | 3 | maximum | ward.D | 0.026 | 10.59, 7.92, 4.93 | 16, 9, 5 | 0.232 |
| 526 | 28 | 3 | maximum | ward.D2 | 0.026 | 10.59, 7.92, 4.93 | 16, 9, 5 | 0.232 |
| 527 | 29 | 3 | maximum | ward.D2 | 0.026 | 10.59, 7.92, 4.93 | 16, 9, 5 | 0.226 |
| 528 | 13 | 2 | maximum | median | 0.025 | 10.59, 7.48 | 7, 23 | 0.079 |
| 529 | 12 | 2 | maximum | median | 0.025 | 10.59, 7.48 | 7, 23 | 0.075 |

**S6 Figure.** Heatmap of gene expression of 30 subjects, clustered based on Parameter set 1 from **S6 Table** (Number of top features = 10, *k* = 2, Distance method = Manhattan, Hierarchical clustering method = ward.D).

**S7 Figure.** Disease-free survival curves were estimated using the non-parametric survival function derived from the Nelson-Aalen estimator. The 30 subjects were clustered using Parameter set 1 from **S6 Table** (Number of top features = 10, *k* = 2, Distance method = Manhattan, Hierarchical clustering method = ward.D). The difference in disease-free survival between clusters was assessed using the Fleming-Harrington (ρ=1, ɣ=1) weighted log-rank test.

(a)

(b)

**S8 Figure.** (a) Disease-free survival curves were estimated using the non-parametric survival function derived from the Nelson-Aalen estimator. The 30 subjects were clustered using Parameter set 1 from **S6 Table**, but with *k* = 4. (b) Pairwise comparison of disease-free survival between the four clusters. Asterisks (*) indicate a statistically significant difference in disease-free survival (p-value ≤ 0.05).

**S7 Table.** Results of clustering analysis using expression profiles of 10 genes identified as important components in our dynamic modeling as features. Parameter set 1, highlighted in red, was chosen for further analysis.

| Parameter set | Number of top features used | Number of clusters (*k*) | Distance method | Hierarchical clustering method | *p*-value | Median disease-free year for each cluster | Number of subjects in each cluster | Silhouette coefficient |
| --- | --- | --- | --- | --- | --- | --- | --- | --- |
| 1 | Set to 10 genes (*RPS6KA2*, *MAPK1*, *CCNE1*, *FOS*, *TNFSF11*, *CDKN1A*, *E2F1*, *RB1*, *CYCS*, and *DUSP5*) | 2 | canberra | ward.D | 0.042 | 10.59, 7.48 | 15, 15 | 0.208 |
| 2 |  | 2 | canberra | complete | 0.042 | 10.59, 7.48 | 15, 15 | 0.208 |
| 3 |  | 2 | canberra | average | 0.042 | 10.59, 7.48 | 15, 15 | 0.208 |
| 4 |  | 2 | canberra | mcquitty | 0.042 | 10.59, 7.48 | 15, 15 | 0.208 |
| 5 |  | 2 | canberra | ward.D2 | 0.042 | 10.59, 7.48 | 15, 15 | 0.208 |
| 6 |  | 2 | euclidean | mcquitty | 0.042 | 10.59, 7.48 | 14, 16 | 0.201 |
| 7 |  | 2 | euclidean | ward.D2 | 0.042 | 10.59, 7.48 | 14, 16 | 0.201 |
| 8 |  | 2 | minkowski | mcquitty | 0.042 | 10.59, 7.48 | 14, 16 | 0.201 |
| 9 |  | 2 | minkowski | ward.D2 | 0.042 | 10.59, 7.48 | 14, 16 | 0.201 |
| 10 |  | 3 | canberra | average | 0.045 | 10.59, NA, 7.48 | 5, 10, 15 | 0.177 |

**S9 Figure.** Heatmap of gene expression of 30 subjects, clustered based on Parameter set 1 from **S7 Table** (*k* = 2, Distance method = Canberra, Hierarchical clustering method = ward.D).

**S10 Figure.** Disease-free survival curves were estimated using the non-parametric survival function derived from the Nelson-Aalen estimator. The 30 subjects were clustered using Parameter set 1 from **S7 Table** (*k* = 2, Distance method = Canberra, Hierarchical clustering method = ward.D). The difference in disease-free survival between clusters was assessed using the Fleming-Harrington (ρ=1, ɣ=1) weighted log-rank test.

**S11 Figure.** (a) Disease-free survival curves were estimated using the non-parametric survival function derived from the Nelson-Aalen estimator. The 30 subjects were clustered using Parameter set 1 from **S7 Table**, but with *k* = 4. (b) Pairwise comparison of disease-free survival among the four clusters. Asterisks (*) indicate a statistically significant difference in disease-free survival (p-value ≤ 0.05).

**References**

1.	Harris LA. Differential equation models for the hormonal regulation of the menstrual cycle: North Carolina State University; 2002.

2.	Scaling AL, Prossnitz ER, Hathaway HJ. GPER mediates estrogen-induced signaling and proliferation in human breast epithelial cells and normal and malignant breast. Horm Cancer. 2014;5(3):146-60. doi: <https://doi.org/10.1007/s12672-014-0174-1>.

3.	Imoto H, Okada M. Signal-dependent regulation of early-response genes and cell cycle: a quantitative view. Curr Opin Syst Biol. 2019;15:100-8. doi: <https://doi.org/10.1016/j.coisb.2019.04.003>.

4.	Tanos T, Sflomos G, Echeverria PC, Ayyanan A, Gutierrez M, Delaloye J-F, et al. Progesterone/RANKL is a major regulatory axis in the human breast. Sci Transl Med. 2013;5(182):182ra55. doi: <https://doi.org/10.1126/scitranslmed.3005654>.

5.	Deng Y, Miki Y, Nakanishi A. Estradiol/GPER affects the integrity of mammary duct-like structures in vitro. Sci Rep. 2020;10(1):1386. doi: <https://doi.org/10.1038/s41598-020-57819-9>.

6.	Legewie S, Bluthgen N, Herzel H. Mathematical modeling identifies inhibitors of apoptosis as mediators of positive feedback and bistability. PLOS Comput Biol. 2006;2(9):e120. doi: <https://doi.org/10.1371/journal.pcbi.0020120>.

7.	Behera MA, Dai Q, Garde R, Saner C, Jungheim E, Price TM. Progesterone stimulates mitochondrial activity with subsequent inhibition of apoptosis in MCF-10A benign breast epithelial cells. Am J Physiol Endocrinol Metab. 2009;297(5):E1089-96. doi: <https://doi.org/10.1152/ajpendo.00209.2009>.
