# Supplementary material for: Integrating dynamic modeling of signaling pathways with subject-specific transcriptomic data to assess breast cancer risk
Source: PLoS One. 2026 Aug 13;21(8):e0355838. doi: 10.1371/journal.pone.0355838 (PMC13472372; doi:10.1371/journal.pone.0355838)
Supplement: Peer review history

# Peer review: Decision Letter 0

**DOI:** 10.1371/journal.pone.0355838.r001

**Authors:** Andre van Wijnen

7 Jun 2026

Dear Dr. Laomettachit,

Thank you for submitting your manuscript to PLOS ONE. After careful consideration, we feel that it has merit but does not fully meet PLOS ONE’s publication criteria as it currently stands. Therefore, we invite you to submit a revised version of the manuscript that addresses the points raised during the review process.

Please submit your revised manuscript by Jul 22 2026 11:59PM. If you will need more time than this to complete your revisions, please reply to this message or contact the journal office at plosone@plos.org. When you're ready to submit your revision, log on to https://www.editorialmanager.com/pone/ and select the 'Submissions Needing Revision' folder to locate your manuscript file.

- A letter that responds to each point raised by the academic editor and reviewer(s). You should upload this letter as a separate file labeled 'Response to Reviewers'.
- A marked-up copy of your manuscript that highlights changes made to the original version. You should upload this as a separate file labeled 'Revised Manuscript with Track Changes'.
- An unmarked version of your revised paper without tracked changes. You should upload this as a separate file labeled 'Manuscript'.

If you would like to make changes to your financial disclosure, please include your updated statement in your cover letter. Guidelines for resubmitting your figure files are available below the reviewer comments at the end of this letter.

If applicable, we recommend that you deposit your laboratory protocols in protocols.io to enhance the reproducibility of your results. Protocols.io assigns your protocol its own identifier (DOI) so that it can be cited independently in the future. For instructions see: https://journals.plos.org/plosone/s/submission-guidelines#loc-laboratory-protocols. Additionally, PLOS ONE offers an option for publishing peer-reviewed Lab Protocol articles, which describe protocols hosted on protocols.io. Read more information on sharing protocols at https://plos.org/protocols?utm_medium=editorial-email&utm_source=authorletters&utm_campaign=protocols.

As the corresponding author, your ORCID iD is verified in the submission system and will appear in the published article. PLOS supports the use of ORCID, and we encourage all coauthors to register for an ORCID iD and use it as well. Please encourage your coauthors to verify their ORCID iD within the submission system before final acceptance, as unverified ORCID iDs will not appear in the published article. *Only*  the individual author can complete the verification step; PLOS staff *cannot*  verify ORCID iDs on behalf of authors.

We look forward to receiving your revised manuscript.

Kind regards,

Andre van Wijnen

Academic Editor

PLOS One

**Journal Requirements:**

When submitting your revision, we need you to address these additional requirements.

1. Please ensure that your manuscript meets PLOS ONE's style requirements, including those for file naming. The PLOS ONE style templates can be found at

https://journals.plos.org/plosone/s/file?id=wjVg/PLOSOne_formatting_sample_main_body.pdf and

https://journals.plos.org/plosone/s/file?id=ba62/PLOSOne_formatting_sample_title_authors_affiliations.pdf

2. Please note that PLOS One has specific guidelines on code sharing for submissions in which author-generated code underpins the findings in the manuscript. In these cases, we expect all author-generated code to be made available without restrictions upon publication of the work. Please review our guidelines at https://journals.plos.org/plosone/s/materials-and-software-sharing#loc-sharing-code and ensure that your code is shared in a way that follows best practice and facilitates reproducibility and reuse.

3. If the reviewer comments include a recommendation to cite specific previously published works, please review and evaluate these publications to determine whether they are relevant and should be cited. There is no requirement to cite these works unless the editor has indicated otherwise.

[Note: HTML markup is below. Please do not edit.]

Reviewers' comments:

Reviewer's Responses to Questions

**Comments to the Author**

1. Is the manuscript technically sound, and do the data support the conclusions?

Reviewer #1: Yes

**********

2. Has the statistical analysis been performed appropriately and rigorously?

Reviewer #1: Yes

**********

3. Have the authors made all data underlying the findings in their manuscript fully available?

The PLOS Data policy

Reviewer #1: Yes

**********

4. Is the manuscript presented in an intelligible fashion and written in standard English?

Reviewer #1: Yes

**********

Reviewer #1: This study presents an interesting systems biology approach to breast cancer risk stratification by combining transcriptomic data from normal breast tissue with a mechanistic ODE-based signaling model. Rather than relying only on static gene-expression differences, the authors used each subject’s RNA-seq profile to personalize simulations of proliferation and apoptosis signaling over a menstrual-cycle-like period, extracted dynamic features from these simulated trajectories, and used them to identify groups with differing future breast cancer risk. The central idea is that hidden differences in signaling dynamics may reveal susceptibility patterns not apparent from gene expression alone. I like this idea; however, I have a few methodological concerns that I believe the authors could clarify to strengthen the interpretation and reproducibility of the work.

Major Comments

1. One methodological point that would benefit from clarification concerns the feature extraction from the simulated ODE trajectories. The manuscript describes “maximum activity” and “minimum activity” as features representing the highest and lowest simulated levels, respectively, yet the methods indicate these were calculated using numpy.argmax and numpy.argmin. As these functions return the index (or time point) at which the maximum/minimum occurs rather than the maximum/minimum value itself, it is unclear whether the intended features were activity magnitudes or timing-based features (e.g., time-to-peak or time-to-nadir). If the latter was intentional, this could indeed be biologically meaningful in the context of dynamic signaling behavior, but the terminology and interpretation should be revised accordingly. If the former was intended, the implementation may need correction (e.g., using numpy.max/numpy.min). The authors should clarify this point and, if necessary, confirm whether the reported clustering and downstream analyses were affected.

2. The manuscript would benefit from a clearer explanation of how the RNA-seq data were used to personalize the ODE model for each subject. It is currently unclear whether gene expression values were used as starting concentrations, to adjust production/degradation rates, or to scale other model parameters. This is important because these choices lead to very different biological interpretations. For example, if a subject has higher MAPK1 RNA expression, does that mean the model starts with more ERK protein, produces ERK faster, or increases ERK signaling activity overall? In addition, some modeled outputs, such as phosphorylated ERK (pERK) and phosphorylated RSK (pRSK), reflect protein activation states that are not directly measured by RNA expression alone. A clearer description of how transcriptomic measurements were translated into these dynamic signaling behaviors would help readers evaluate the biological validity and reproducibility of the model.

Minor Comments:

1. Lines 82–87 would benefit from clearer wording and additional context. The statement that breast tissue adjacent to tumors displays “unique biological characteristics” is vague—please specify which characteristics are being referenced and provide appropriate supporting citations. In addition, the discussion of normal breast tissue expression subtypes sharing features with the claudin-low phenotype (lines 86–87) is somewhat confusing and could be clarified to better explain its relevance to breast cancer susceptibility.

2. For the reported differences in gene expression between susceptible and non-susceptible groups (e.g., MAPK1, RPS6KA2, CCND1, RB1, CDKN1A, SKP2, and CYCS), please specify the statistical test used, along with the corresponding p-values (or adjusted p-values) and effect sizes where applicable. The statement that CYCS was “less statistically significant” is particularly unclear without quantitative reporting.

**********

what does this mean?). If published, this will include your full peer review and any attached files.

If you choose “no”, your identity will remain anonymous but your review may still be made public.

**Do you want your identity to be public for this peer review?**  For information about this choice, including consent withdrawal, please see our Privacy Policy

Reviewer #1: No

**********

[NOTE: If reviewer comments were submitted as an attachment file, they will be attached to this email and accessible via the submission site. Please log into your account, locate the manuscript record, and check for the action link "View Attachments". If this link does not appear, there are no attachment files.]

To ensure your figures meet our technical requirements, please review our figure guidelines: https://journals.plos.org/plosone/s/figures

You may also use PLOS’s free figure tool, NAAS, to help you prepare publication quality figures: https://journals.plos.org/plosone/s/figures#loc-tools-for-figure-preparation.

NAAS will assess whether your figures meet our technical requirements by comparing each figure against our figure specifications.

# Author comment: Author response to Decision Letter 1

**DOI:** 10.1371/journal.pone.0355838.r002

3 Jul 2026

The Response to Reviewers document has been uploaded as a separate file with the submission.

Attachment Submitted filename: Response_to_Reviewers_PLOSONE_2026_r1.pdf

# Editor report: Decision Letter 1

**DOI:** 10.1371/journal.pone.0355838.r003

**Authors:** Andre van Wijnen

28 Jul 2026

Integrating dynamic modeling of signaling pathways with subject-specific transcriptomic data to assess breast cancer risk

PONE-D-26-04978R1

Dear Dr. Laomettachit,

We’re pleased to inform you that your manuscript has been judged scientifically suitable for publication and will be formally accepted for publication once it meets all outstanding technical requirements.

Within one week, you’ll receive an e-mail detailing the required amendments. When these have been addressed, you’ll receive a formal acceptance letter and your manuscript will be scheduled for publication.

An invoice will be generated when your article is formally accepted. Please note, if your institution has a publishing partnership with PLOS and your article meets the relevant criteria, all or part of your publication costs will be covered. Please make sure your user information is up-to-date by logging into Editorial Manager at Editorial Manager® and clicking the ‘Update My Information' link at the top of the page. For questions related to billing, please contact billing support.

If your institution or institutions have a press office, please notify them about your upcoming paper to help maximize its impact. If they’ll be preparing press materials, please inform our press team as soon as possible -- no later than 48 hours after receiving the formal acceptance. Your manuscript will remain under strict press embargo until 2 pm Eastern Time on the date of publication. For more information, please contact onepress@plos.org.

Kind regards,

Andre van Wijnen

Academic Editor

PLOS One

Additional Editor Comments (optional):

Editorial Comments:

The authors have adequately addressed the previous comments.

Reviewers' comments:

# Editor report: Acceptance letter

**DOI:** 10.1371/journal.pone.0355838.r004

**Authors:** Andre van Wijnen

PONE-D-26-04978R1

PLOS One

Dear Dr. Laomettachit,

I'm pleased to inform you that your manuscript has been deemed suitable for publication in PLOS One. Congratulations! Your manuscript is now being handed over to our production team.

At this stage, our production department will prepare your paper for publication. This includes ensuring the following:

* All references, tables, and figures are properly cited

* All relevant supporting information is included in the manuscript submission,

* There are no issues that prevent the paper from being properly typeset

You will receive further instructions from the production team, including instructions on how to review your proof when it is ready. Please keep in mind that we are working through a large volume of accepted articles, so please give us a few days to review your paper and let you know the next and final steps.

Lastly, if your institution or institutions have a press office, please let them know about your upcoming paper now to help maximize its impact. If they'll be preparing press materials, please inform our press team within the next 48 hours. Your manuscript will remain under strict press embargo until 2 pm Eastern Time on the date of publication. For more information, please contact onepress@plos.org.

You will receive an invoice from PLOS for your publication fee after your manuscript has reached the completed accept phase. If you receive an email requesting payment before acceptance or for any other service, this may be a phishing scheme. Learn how to identify phishing emails and protect your accounts at https://explore.plos.org/phishing.

If we can help with anything else, please email us at customercare@plos.org.

Thank you for submitting your work to PLOS ONE and supporting open access.

Kind regards,

PLOS ONE Editorial Office Staff

on behalf of

Dr. Andre van Wijnen

Academic Editor

PLOS One
